# Inflammation indices in association with periodontitis and cancer

**DOI:** 10.1111/prd.12612

**Published:** 2024-09-24

**Authors:** Kay‐Arne Walther, Sabine Gröger, Jonas Adrian Helmut Vogler, Bernd Wöstmann, Jörg Meyle

**Affiliations:** ^1^ Department of Periodontology, Dental Clinic Justus Liebig University of Giessen Giessen Germany; ^2^ Department of Prosthodontics, Dental Clinic Justus Liebig University of Giessen Giessen Germany; ^3^ Department of Orthodontics, Dental Clinic Justus Liebig University of Giessen Giessen Germany; ^4^ Department of Periodontology, Dental Clinic University of Bern Bern Switzerland

**Keywords:** cancer, delta neutrophil index, inflammation indices, inflammation markers, lymphocyte to monocyte ratio, neutrophil to lymphocyte ratio, periodontitis, platelet distribution width, platelet to lymphocyte ratio, plateletcrit, red blood cell distribution width, systemic immune inflammation index

## Abstract

Inflammation is a complex physiological process that plays a pivotal role in many if not all pathological conditions, including infectious as well as inflammatory diseases, like periodontitis and autoimmune disorders. Inflammatory response to periodontal biofilms and tissue destruction in periodontitis is associated with the release of inflammatory mediators. Chronic inflammation can promote the development of cancer. Persistence of inflammatory mediators plays a crucial role in this process. Quantification and monitoring of the severity of inflammation in relation to cancer is essential. Periodontitis is mainly quantified based on the severity and extent of attachment loss and/or pocket probing depth, in addition with bleeding on probing. In recent years, studies started to investigate inflammation indices in association with periodontal diseases. To date, only few reviews have been published focusing on the relationship between blood cell count, inflammation indices, and periodontitis. This review presents a comprehensive overview of different systemic inflammation indices, their methods of measurement, and the clinical applications in relation to periodontitis and cancer. This review outlines the physiological basis of inflammation and the underlying cellular and molecular mechanisms of the parameters described. Key inflammation indices are commonly utilized in periodontology such as the neutrophil to lymphocyte ratio. Inflammation indices like the platelet to lymphocyte ratio, platelet distribution width, plateletcrit, red blood cell distribution width, lymphocyte to monocyte ratio, delta neutrophil index, and the systemic immune inflammation index are also used in hospital settings and will be discussed. The clinical roles and limitations, relationship to systemic diseases as well as their association to periodontitis and treatment response are described.

## 1 INTRODUCTION

Periodontitis, characterized by progressive destruction of the tooth‐supporting structures, is a chronic inflammatory disease with a multifactorial etiology^1^ linked to the patient's individual oral microbiome and immune response.^2^ The manifestation of periodontitis depends on a dysbiotic biofilm,^3^ whereas microbial factors alone are insufficient to induce onset of the disease.^4^, ^5^, ^6^ Dysregulation of the immune response in periodontitis plays a major role in pathogenesis.^7^ The susceptibility to periodontitis is influenced by genetic^8^, ^9^, ^10^ and lifestyle factors, particularly smoking and poor oral hygiene, which facilitate the expression of bacterial pathogenicity.^11^

Progression of periodontitis results primarily from the host's response to the microbial biofilm, mediated by neutrophils, monocytes, macrophages, and T‐ and B‐lymphocytes.^4^ Neutrophils, being the most abundant leukocytes, are central to inflammatory pathways, bridging innate and adaptive immunity and are often seen as a double‐edged sword in immune response.^12^ The involvement of T‐ and B‐lymphocytes in the pathogenesis of periodontitis has been extensively studied. The activation of various T cells, monocytes, and macrophages leads to the production of numerous pro‐inflammatory cytokines, culminating in the progressive destruction of the tooth‐supporting structures.^4^

**FIGURE 1:**
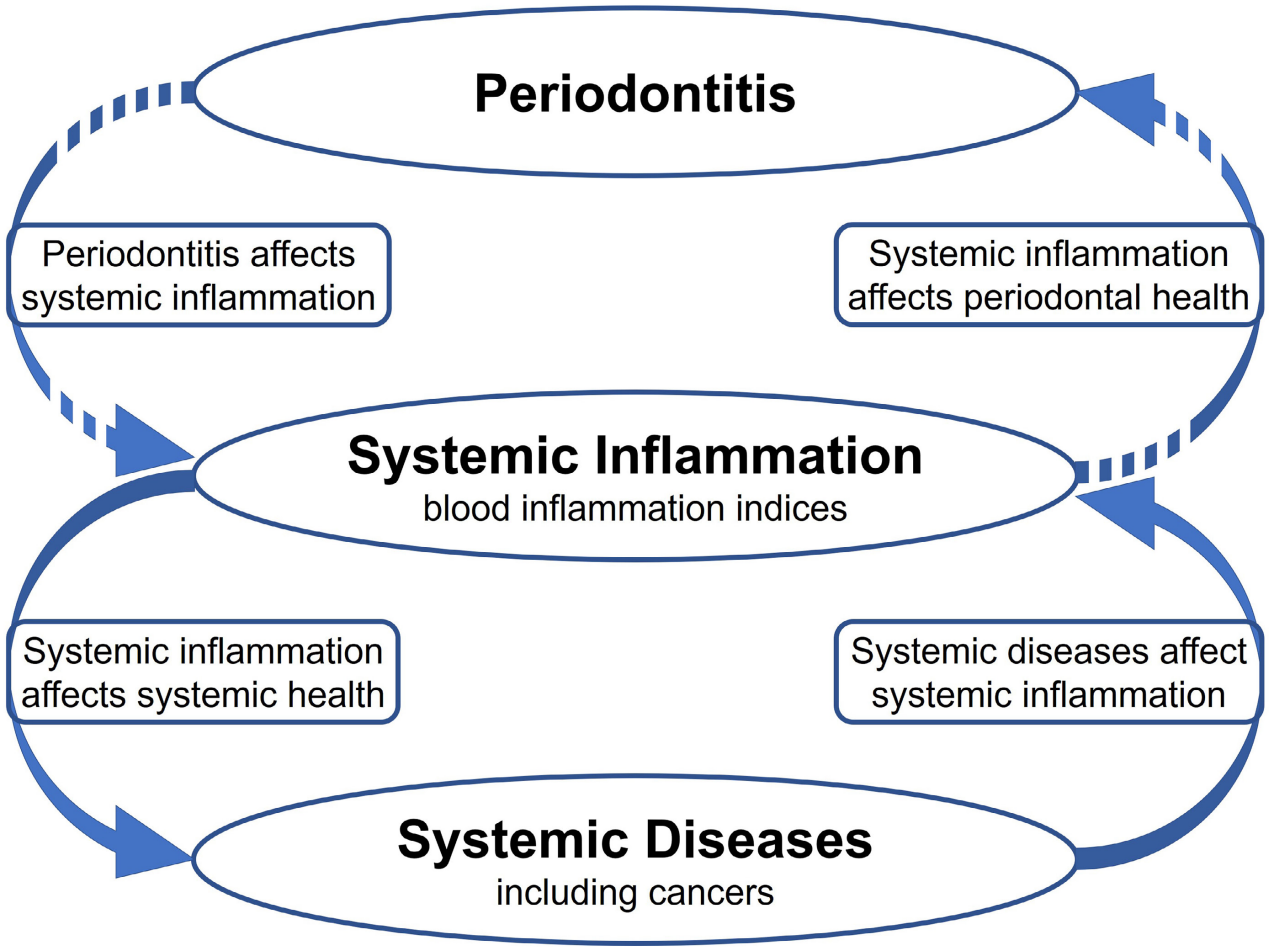
Inflammation resulting from periodontitis may extend to a systemic level, thereby influencing the subject's overall inflammatory burden and systemic health.^16^ Conversely, systemic diseases can influence systemic inflammation, which in turn may affect periodontal health.^17^ Patients suffering from periodontitis may have elevated levels of circulating systemic inflammatory markers, including higher inflammation indices. Figure modified after Cecoro et al.^21^

The dentogingival epithelial surface, encompassing the pocket epithelium in direct contact with the subgingival biofilm, acts as a crucial interface where local inflammation can affect systemic health. The dentogingival junction is a semi‐permeable barrier that permits the entry of the numerous bacteria, whether planktonic or in biofilm, into the bloodstream, known as bacteremia. Under conditions where microbial loads are minimal and gingival tissues are healthy, such bacteremia typically constitutes minimal threat to host tissues and shows low systemic impact.^13^, ^14^ This is attributed to the activity of the innate immune system in dentate mammals, which are well able to respond to and prevent the entry of bacteria through the dentogingival junction. Approximately, the size of an adult's palm is the area of inflamed epithelial surface which persists during severe periodontitis.^15^ As a result, locally produced pro‐inflammatory mediators such as interleukins, tumor necrosis factor alpha, and prostaglandins may enter the circulation, contributing to an elevated inflammatory load at a systemic level and potentially affecting remote organs.^3^, ^13^, ^16^ Conversely, systemic inflammation can also influence periodontal health (Figure 1).^17^

Diagnosing periodontitis during its active phase and identifying individuals' risk of developing a severe form are challenges for clinicians and researchers. Advances in diagnostic techniques for periodontal diseases tend to methods for identification and quantification of the periodontal risk in patients through various biomarkers that can be detected in saliva, gingival crevicular fluid, or serum.^18^, ^19^ Studies have shown that patients with periodontitis, influenced by the composition of the oral microbiome,^20^ exhibit higher systemic levels of various inflammatory markers, and this indicates increased low‐grade systemic inflammation.^18^, ^21^, ^22^, ^23^, ^24^ Blood inflammation indices such as neutrophil to lymphocyte ratio (NLR),^25^, ^26^, ^27^, ^28^, ^29^, ^30^, ^31^, ^32^, ^33^, ^34^ platelet to lymphocyte ratio (PLR),^35^, ^36^, ^37^, ^38^, ^39^, ^40^ and lymphocyte to monocyte ratio (LMR)^41^, ^42^, ^43^, ^44^, ^45^ have been demonstrated as being helpful in diagnosis and prognosis of several chronic inflammatory diseases, including cardiovascular diseases, diabetes mellitus, chronic lung disease, and various cancers. However, these have hardly been used in the periodontology.

In the pathophysiology of most hematopoietic and solid malignancies, inflammation plays a central role.^46^ Tumorigenesis can be induced by chronic inflammation and as a pathological response to persisting infection, immune disorders, and aging in susceptible individuals. The initiation of tumor formation activates “cancer‐elicited inflammation” based on a chemokines and pro‐inflammatory cytokine storm. This cascade leads to the recruitment of immune cells, the induction of angiogenesis, and a transition to the promoting phase.^46^ Stimulation of tumor‐associated macrophages to secrete IL‐1β and activation of tumor‐associated neutrophils contribute to metastatic progression and enhance systemic neutrophilic inflammation. This perspective underscores the potential of systemic inflammation indices as simple, economical, and easily accessible tools to indicate systemic inflammation^47^ especially to study cancer‐related inflammation and these could be valuable indicators of prognosis for different cancers.^48^, ^49^ Numerous meta‐analyses have investigated the prognostic significance of the discussed inflammation indices in this review in relation to various cancers.

A recently published review by Baima et al.^50^ provides a good overview of the pathogenic mechanisms between periodontitis and different types of cancer. However, the relationship between different systemic inflammation indices and periodontitis, which could serve as potential markers of systemic inflammation, especially as risk for various forms of cancer, remains poorly understood and warrants further investigation. To date, no study has investigated inflammation indices in periodontitis patients with cancer. Therefore, this review discusses the current literature of systemic inflammatory indices in relation to periodontitis and cancer, including blood values for NLR, PLR, platelet distribution width (PDW), plateletcrit (PCT), red blood cell distribution width (RDW), LMR, delta neutrophil index (DNI), and systemic immune inflammation index (SII) and to assess their usefulness in diagnosis, screening, and predicting the risk of developing severe diseases like cancer.

## 2 BLOOD INFLAMMATION MARKERS

Currently, periodontitis is diagnosed using local clinical and radiographic criteria, with minimum use of diagnostic tools that evaluate systemic biomarkers.^51^ Notably, during the 2017 World Workshop on the Classification of Periodontal and Peri‐Implant Diseases and Conditions, it became evident that integration of inflammation markers into the diagnosis and monitoring of periodontitis is needed.^52^ In medical practice, a full blood count or hemogram is commonly used to detect the presence of infection or inflammation. This raises the question of whether periodontitis influences hematological parameters, including the counts of white blood cells (WBC), red blood cells, and platelets.^53^, ^54^ In particular, the components of the most frequently used inflammation indices like neutrophils, platelets, and lymphocytes play a crucial role in innate and adaptive immunity.^55^

### 2.1 Neutrophils

Neutrophils are the most abundant WBC and are crucial in the innate immune system as initial answer in the immune response.^56^ In healthy individuals, in the periodontal tissues, neutrophils are typically located in the superficial layers of the epithelium and at the base of the gingival sulcus.^57^ Microscopically, neutrophils are present in the intercellular spaces of the permeable junctional epithelium and along tooth surfaces, with their distribution being highest at the base of the gingival sulcus, decreasing toward the entrance of the gingival crevice. Neutrophils are essential for protecting periodontal tissues against microbial challenges. Over 50 years ago, Schiött and Löe^58^ described, that in the presence of periodontal disease, the migration rate of neutrophils into the oral cavity increases. Since then, numerous studies demonstrated that their hyperactive state could be responsible for periodontal tissue damage.^59^, ^60^ If neutrophils are overloaded by any biofilm, lysosomal enzymes and reactive oxygen species (ROS) can cross into the extracellular matrix and increase the risk of host tissue damage and as such they act as a double‐edged sword.^61^ Neutrophils also extend their antimicrobial activity beyond their lifespan through neutrophil extracellular traps (NETs) formation, a process known as NETosis.^62^ During NETosis, neutrophil nuclei swell, chromatin dissolves, and DNA is unwound and is exported out of the cells with histones and proteins from granules and cytosol. NETs trap and immobilize pathogens in the web of DNA, preventing their spread and preventing direct antimicrobial activity through proteolytic and ROS interactions in the extracellular matrix.^62^, ^63^, ^64^

*Aggregatibacter actinomycetemcomitans* (*A. actinomycetemcomitans*) as well as different virulence factors from *Porphyromonas gingivalis* (*P. gingivalis*) compromise numerous neutrophil functions. These gram‐negative bacteria secure their access to nutritional proteinaceous resources and as such facilitate the perpetuation of inflammation and evade microbial destruction. Neutrophils play a role in the progression of periodontitis due to impaired antimicrobial activity, including a dysregulation of immune tolerance, altered neutrophil apoptosis, and disrupted mechanisms that mediate the resolution of inflammation. Nevertheless, possibly after bacterial encounters, these manipulations of neutrophils can lead to divergent outcomes, manifesting either pro‐inflammatory or anti‐inflammatory effects.^65^, ^66^

An increase in peripheral blood neutrophil count in patients with periodontitis was observed in several studies.^60^, ^67^ Studies have analyzed changes in neutrophil counts in peripheral blood to identify and monitor periodontitis, similar to diagnosis and monitoring of systemic diseases. It could be demonstrated, that patients with generalized aggressive periodontitis (GAgP), who are otherwise healthy, exhibited decreased neutrophil counts following periodontal therapy.^68^ Overall, systemic neutrophil counts are an essential component as part of inflammatory indices for any disease and also periodontitis.

### 2.2 Platelets

Platelets (also known as thrombocytes) are small, anucleate cells in the blood that are essential for hemostasis, and also play a significant role in wound healing and the inflammatory processes.^69^, ^70^ They are increasingly recognized for their role in both the initiation and progression of periodontitis.

Prospective epidemiological studies consistently demonstrated an association between elevated platelet counts, including their activation levels and inversely correlated mean volume and the subsequent manifestation and progression of fatal coronary heart disease.^71^ Additionally, thrombocytosis has been identified as a prognostic marker for conditions such as venous thromboembolism, stroke, cancer, and ultimately increased mortality rates.^71^, ^72^, ^73^

Periodontitis has been linked to increased serum levels of IL‐6.^74^ This cytokine has the capability to stimulate hepatic thrombopoietin production and subsequently thrombopoiesis, leading to increased platelet counts (thrombocytosis).^75^, ^76^, ^77^ Additionally, platelets share a signaling pathway with WBC, that maintains differentiation of the cells, highlighting their significant role in immunity and microbial defense.^36^, ^78^, ^79^ Platelets express various antigen recognition molecules and can directly interact with microbes during bacteremia, resulting in an increase in their number and activity.^80^, ^81^ Platelets can interact with periodontal pathogens like *P. gingivalis*.^82^ This interaction can lead to a formation of platelet‐bacteria aggregates, serving as a target for neutrophils for phagocytosis and NETosis to eliminate bacteria facilitated by these bundles.^83^ Furthermore, platelets are involved in tissue repair processes due to their role in angiogenesis and wound healing. In the periodontium, these capabilities of platelets can be harnessed therapeutically, for example, in the application of platelet‐rich plasma applications to promote periodontal regeneration.^84^

Several studies documented a significant elevation in platelet counts among patients with periodontitis.^85^, ^86^, ^87^, ^88^, ^89^ Studies also observed no difference in platelet levels when comparing patients with severe periodontitis to healthy individuals.^37^, ^90^, ^91^ This suggests that the activation and function of platelets, rather than their absolute counts, may play a critical role in the pathogenesis of periodontitis and in the systemic effects of periodontitis.^92^ One of the largest cohort studies including 5197 South Korean subjects demonstrated elevated platelet counts in periodontitis patients. The association between severe periodontitis and platelet counts has been demonstrated to be stronger than the associations of age and smoking status with platelet counts in the same statistical models.^89^ Studies that evaluated the results of nonsurgical periodontal therapy observed a decrease in both the number of platelets and their hyperreactivity in peripheral circulation.^68^, ^92^, ^93^, ^94^ Furthermore, the inhibition of platelet activation has been found to contribute to the reduction of periodontal inflammation.^95^

In summary, platelets play a multifactorial role in the inflammatory processes, and therefore, are import parameters in inflammatory indices. In particular, this review not only includes the platelet count in the PLR but also the PCT and PDW are indices specific to platelet proliferation kinetics and morphology.

### 2.3 Lymphocytes

Lymphocytes are crucial components of the adaptive immune system and play a significant role in all aspects of immune responses, not only infections but also in cancer. B and T cells, recognize and respond to specific antigens of both, all kinds of pathogens and to cellular antigens. T cells, once activated, show plasticity and can “rotate” into various subsets (like Th1, Th2, Th17, Tfh, and regulatory T cells [Treg]), all playing a distinct role in exerting and modulating the immune response. B cells differentiate into memory B cells or plasma cells that produce antibodies. The suppression of T‐cell activation in severe periodontitis can result from interactions with various periodontal pathogens.^96^, ^97^ Th1 cells mainly express the transcription factors STAT‐4 and T‐bet, they predominantly produce the cytokines IL‐12 and interferon gamma (IFN‐γ). Their differentiation is induced by IL‐12. Conversely, Th2 cells are marked by the expression of transcription factors GATA‐3 and STAT‐6, and the production of the cytokines IL‐4, IL‐5, IL‐6, IL‐10, and IL‐13.^98^ Th1 and Th2 cells respond to different stimuli and are associated with various outcomes in infectious and inflammatory diseases. In some studies, T‐cell subset cytokine profiles indicate that the number of Th1 cells correlates with stable lesions, while Th2 cells are associated with progressive lesions,^99^, ^100^, ^101^ suggesting a link between Th2 cells and more severe lesions. However, other studies have shown a predominance of Th1 cells in gingival tissues from periodontitis patients, associated with inflammation and disease severity.^102^, ^103^ Remarkably, one study reported that both Th1 and Th2 responses are similarly upregulated in periodontal lesions.^104^

Th17 cells expressing the transcription factor RORγt produce predominantly the cytokines IL‐17 and IL‐21. They require IL‐6, IL‐21, IL‐23, and TGF‐β for their development and maintenance. Th17 cells play a crucial role in the defense against extracellular pathogens and fungi, as well as in promoting inflammation.^105^ Recent studies linked Th17 cells to various inflammatory and autoimmune diseases.^106^, ^107^, ^108^ Interestingly, increased Th17 cell infiltration in periodontal lesions was demonstrated. This supports the presumed association with periodontitis.^109^, ^110^ Furthermore, studies investigated the relationship between *A. actinomycetemcomitans* infection and systemic immune responses associated with Th17 cells.^111^ It has been demonstrated that oral infection with *A. actinomycetemcomitans*, through bacteremia, can contribute up to 20% of the HACEK bacteria group, which is a significant risk factor for infective endocarditis.^112^ In addition, the differentiation of CD4+ subtypes (in particular Th1 and Th17)^113^ can be stimulated through the interaction of lipopolysaccharide (LPS) and toll‐like receptors (TLRs), which activates the NF‐κB pathway.^114^, ^115^ Zhang et al.^116^ demonstrated that LPS from *P. gingivalis* could promote Th17 cell differentiation directly by upregulating TLR‐2 expression on the T‐cell membrane.

Tregs are a suppressive lymphocyte subset that maintain downregulation of activation, proliferation, and function be specific. Tregs are characterized by the secretion of the cytokines TGF‐β and IL‐10 and express the transcription factor Foxp3. Also, Tregs express higher levels of certain cell surface molecules like GITR, CD25, and CTLA‐4, compared to naive Th cells. They play a critical role in maintaining immune homeostasis by modulating the intensity and direction of both adaptive and innate immunity, including inflammation.^117^, ^118^ Several studies demonstrated high levels of Tregs in periodontitis lesions.^119^, ^120^, ^121^ Another study reported a reduction in Foxp3^+^CD25^+^ cells in such lesions.^122^

A special subset of lymphocytes are γδT cells, which are mainly located in the epithelial barrier of the gingiva. Within these again, subgroups exist: γδT cells exhibit different immunological functions, including immunoregulatory capacities, cytolytic activity, and fast secretion of inflammatory cytokines.^123^ Investigations in humans and mice have demonstrated that γδT cells can exert both antitumor and pro‐tumor activities.^124^, ^125^ The dualistic nature of these roles may be attributed to the tendency of murine γδT cells to produce IL‐17, a cytokine implicated in promoting cancer development.^126^ In contrast, human γδT cells demonstrate potent cytotoxic capabilities and IFN‐γ production, suggesting a potential antitumor role.^127^ A recent review which summarizes the importance of the γδT cells in the oral epithelium during carcinogenesis is further explained in a paper published by Hovav and Wilensky.^128^ The differentiation of Th cells in periodontitis is at least partially attributable to pathogens and their virulence factors. The results of numerous studies suggest a protective function of lymphocytes, while more recent studies underscore the complexity of lymphocyte immunity in periodontitis, partly due to the presence of T‐cell subsets that suppress the function of pro‐inflammatory T‐cell populations.^129^ In conclusion, lymphocytes and their subgroups give us an important insight into the pathogenesis of periodontitis. In future, inflammation indices should also include lymphocyte subgroups.

## 3 INFLAMMATION INDICES—SELECTION AND CHARACTERISTICS OF THE INCLUDED STUDIES

To date, there are no comprehensive reviews existing, which focus on the relationship between inflammatory indices, periodontitis, and cancer. The following indices are based on hematological parameters derived from a standard full blood count. Among the indices which have been described in the available literature those were selected where an association with periodontitis has been reported.

A structured electronic search was conducted in May 2023 of the following databases: PubMed/MEDLINE and Web of Science. MeSH and Emtree terms were used. The most actual electronic search was performed on all databases on October 30, 2023.

The search strategy included the terms “inflammation indices,” “inflammation markers,” “neutrophil to lymphocyte ratio,” “platelet to lymphocyte ratio,” “platelet distribution width,” “plateletcrit,” “red blood cell distribution width,” “lymphocyte to monocyte ratio,” “delta neutrophil index,” “systemic immune inflammation index,” “cancer,” “periodontitis,” and “periodontal” as free text words, along with MeSH or Emtree terms (if available), synonyms, singular as well as plural forms and abbreviations (NLR, PLR, PDW, PCT, RDW, RDW‐CV, RCDW, RDW‐SD, LMR, DNI, and SII). Furthermore, filters for “Humans” and “English” were used. We excluded editorials, conference abstracts, case reports, mechanistic articles, and animal studies. Consequently, 11 case–control studies, 4 cross‐sectional studies, and 1 meta‐analysis were included. These studies will be discussed below. An overview with the important characteristics and results is presented in Table 1.

**TABLE 1:** Systemic inflammation indices in association with periodontitis.

Parameters	Autor, year, country, and reference	Analysis	Study design	Number of participants, gender, and age	Number of diagnosed participants	Examiner calibration	Smoking status	Significant difference in age between groups	Significant difference in gender between groups	Main results	Conclusion
**Neutrophil to lymphocyte ratio (NLR)**	Acharya et al., 2019, India^90^	Automated hematology analyzer (pocH‐100i; Sysmex)	Case–control and prospective cohort	*n* = 60 (30 males, 30 females, mean age: P 45.08 years and H 39.6 years)	30 CP^130^ versus 30 H	No	Not reported	Not reported	Not reported	NLR of pretreatment with CP: 1.90 (SD = ±0.5), NLR of posttreatment: 1.48 (SD = ±0.4), *p* < 0.0001, NLR of H: 1.86 (SD = ±0.81); NLR in pre‐ and post‐treated patients was not associated to local periodontal parameters; cutoff point by ROC for pre‐ versus post‐treated patients = 1.546 (sensitivity = 0.756, specificity = 0.756, AUC = 77.5%)	Significant difference between pre‐ and posttreatment CP patients
	Bhattacharya et al., 2022, India^131^	Not reported	Cross‐sectional	*n* = 80 (40 males, 40 females, mean age P 41.8 years and H 36.93 years)	40 P^132^ versus 40 H	Yes	Only nonsmokers	+	−	NLR of P: 2.48 (SD = ±1.17), NLR of H: 2.02 (SD = ±0.5), significant difference (*p* = 0.013)	NLR is associated with P
	Çetin Özdemir et al., 2022, Turkey^133^	Automated hematology analyzer (company not reported)	Case–control	*n* = 77 (28 males, 46 females, mean age 36 years)	22 stage III grade A P^52^ versus 26 gingivitis versus 26 H^134^	Yes	Only nonsmokers	+	+	NLR of H: 1.85 (SD = ±0.67), NLR of gingivitis: 1.96 (SD = ±0.78), NLR of P: 2.22 (SD = ±0.85), no association between NLR and gingivitis and P (*p* = 0.156); correlation for CAL, mGI, plaque index, and NLR but not for BOP (Spearman's correlation test)	NLR is not associated with gingivitis or P
	Doğan et al., 2015, Turkey^135^	Not reported	Case–control	*n* = 180 (69 males, 111 females, mean age not reported)	P versus non‐P (number and classification not reported); categorized as H (*n* = 28), type 2 diabetes (*n* = 75), hyperlipidemia (*n* = 99), obese (*n* = 119), and (pre‐ and post‐) menopause (*n* = 37)	Yes, but weighted kappa coefficients for intra‐examiner reliability of one periodontist not reported	Smokers and nonsmokers, significance not reported	Not reported between periodontal groups	Not reported between periodontal groups	NLR in H: 2.82 (SD = ±2.27), NLR in type 2 diabetes: 2.48 (SD = ±1.56), and NLR in hyperlipidemia: 2.41 SD = ±1.43, NLR in obese: 2.43 (SD = ±1.49), NLR in menopause: 2.57 (SD = ±1.36); Hyperlipidemic group (*n* = 99): NLR was significant higher in P (NLR: 2.56, SD = ±1.50) than in non‐P (NLR: 2.05, SD = ±1.31), *p* < 0.05	NLR is not associated with type 2 diabetes, hyperlipidemia, obese, or menopause, but associated in hyperlipidemic patients with P
	Lu et al., 2021, China^91^	Automated hematology analyzer (Sysmex XS ‐ 1000)	Case–control	*n* = 505 (205 males, 300 females, mean age 27 years)	372 generalized AgP^136^ versus 133 H	Yes	Only nonsmokers	−	−	NLR of AgP: 2.34 (SD = ±1.11), NLR of H: 1.84 (SD = ±0.85), significant difference between H and AgP (*p* < 0.001); AgP showed significantly higher proportions in NLR 2–3 and NLR ≥3 subgroups; AgP group: 1 unit increment of NLR was associated with an increase in PPD by 0.41 mm (CI = 0.25–0.56), BOP by 0.26 (CI = 0.15–0.37), and CAL by 0.57 mm (CI = 0.34–0.80); Diagnostic ability of NLR by ROC plot: no significant differences, AUC = 0.73, (CI = 0.68–0.79), cutoff point = 1.92, sensitivity = 65.6%, specificity = 68.7%; Saturation threshold effect of NLR (spline smoothing fitting): 1. linear analysis: 0.1 unit increment of NLR, risk of AgP increases by 10.5%, 2. nonlinear analysis: turning point value of NLR = 3, → NLR <3: risk of AgP increased by 20.6% in patients for each 0.1 unit increment of NLR (adjusted OR = 3.06, CI = 1.91–4.98) → NLR >3: OR did not increase with increment of NLR (adjusted OR = 0.94, CI = 0.56–1.57) → *p*‐value for likelihood ratio test of the models = 0.014, demonstrating a nonlinear relationship between NLR and risk of AgP	NLR is highly associated with generalized AgP
	Mishra et al., 2022, India^137^	Automated hematology analyzer (Sysmex XN ‐ 1000)	Case–control	*n* = 630 (324 males, 306 females, mean age 34 years)	315 generalized stage III grade C P^52^ versus 315 H^134^	Yes	Only nonsmokers	−	−	NLR of P: 2.74 (IQR = 2.4–3.28), NLR of H: 2.13 (IQR = 1.81–2.47), significant difference between H and P patients (*p* < 0.0001), ROC cutoff value >2.44, MLRA: high significant association of P with NLR (OR = 9.65, CI 6.59–14.13, *p* < 0.0001) after adjusting for BMI, WBC count, and oral hygiene habits; predictive validity in discriminating P patients from H individuals as depicted by AUC = 0.788 (CI 0.754–0.819, *p* < 0.0001); NLR: sensitivity = 73.97% (CI 68.75–78.72%), specificity = 80.0% (CI 75.15–84.27%), and diagnostic accuracy = 76.98% (CI 73.49–80.21%)	NLR is highly associated with P
	Mishra et al., 2022, India^138^	Automated hematology analyzer (Sysmex XN ‐ 1000)	Case–control	*n* = 148 (80 male, 68 females, mean age 31 years)	108 generalized stage III grade C P^52^ versus 40 H	Yes	Only nonsmokers	−	−	NLR of P: 2.84 (SD = ±1.18), NLR of H: 2.10 (SD = ±1.08), significant difference between H and P patients (*p* < 0.0001); NLR is significant positive associated with mean CAL, mean PPD, mean BOP, mean PI, and mean mGI; ROC analysis yielded cutoff values of >2.15 in predicting risk of P, predictive validity as indicated by AUC = 0.743 (CI 0.627–0.779, *p* < 0.0001); based on these cutoff values: OR of *p* = 11.43 (CI 4.80–27.19, *p* < 0.0001) with every 0.1 unit increment in NLR; Logistic regression analysis (age, male gender, and BMI as explanatory variables and NLR values as dependent variables): age was a significant predictor of difference (*p* = 0.02), but not male gender (*p* = 0.49) and BMI (*p* = 0.72); NLR: sensitivity = 76.85% (CI 67.75%–84.42%), specificity = 77.50% (CI 61.54%–89.16%) and diagnostic accuracy = 77.02% (CI 69.40%–83.53%)	NLR is highly associated with P
	Temelli et al., 2018, Turkey^139^	Selective coronary angiography with contrast agent by means of Judkins technique in multiple projections; clinical chemistry analyzer (AU5800; Beckmann Coulter)	Case–control	*n* = 77 (47 males, 30 females, mean age Group 1 = 59.5 years, Group 2 = 57.5 years, Group 3 = 50 years, Group 4 = 49 years)	41 P^136^ versus 36 non‐P (gingivitis^140^); divided into group 1 = CAD with P (*n* = 20), group 2 = CAD without P (*n* = 20), group 3 = non‐CAD with P (*n* = 21), and group 4 = non‐CAD without P (*n* = 16)	Yes, of periodontists and cardiologists	Discussed smoking and found no significant differences	+	−	NLR of group 1: 2, NLR of group 2: 2, NLR of group 3: 2, NLR of group 4: 1,5; no significant differences between groups	NLR is not associated to patients with/without CAD and/or P
	Torrungruang et al., 2018, Thailand^141^	Automated hematology analyzer (Cell‐Dyn Ruby System; Abbott Diagnostics)	Cross‐sectional	*n* = 2036 (gender and age not reported)	1555 normoglycemic, 331 IFG and 150 diabetes patients^142^; divided into: 365 no/mild P, 1058 moderate P, and 613 severe P^143^	Yes	Nonsmokers, former smokers, and current smokers with significant differences	+	+	NLR of normoglycemic: 1.4 (IQR = 1.1–1.8), NLR of IFG: 1.4 (IQR = 1.1–1.8), and NLR of diabetes: 1.4 (IQR = 1.1–1.8), *p* = 0.212; NLR of no/mild P: 1.4 (IQR = 1.1–1.8), NLR of moderate P: 1.4 (IQR = 1.1–1.8), and NLR of severe P: 1.5 (IQR = 1.2–1.9), *p* = 0.057; Comparison between no/mild/moderate P versus severe P was more likely to have higher NLR (*p* = 0.019); after controlling for P severity, NLR was not associated with diabetes (*p* > 0.05)	NLR is not associated to patients with/without diabetes and with/without P
**Delta neutrophil index (DNI)**	Çetin Özdemir et al., 2022, Turkey^133^	Automated hematology analyzer (company not reported)	Case–control	*n* = 77 (28 males, 46 females, and mean age 36 years)	26 gingivitis versus 22 stage III grade A P^52^ versus 26 H^134^	Yes	Only nonsmokers	+	+	DNI of H: 0.23 (SD = ±0.07), DNI of gingivitis: 0.33 (SD = ±0.11), DNI of P: 0.46 (SD = ±0.22), DNI was significant associated between groups (*p* < 0.001); no correlations for CAL, BOP, mGI, plaque index, and DNI (Spearman's correlation test), ROC analysis: 0.25 cutoff value for DNI in diagnosis of periodontal disease (sensitivity = 91% and specificity = 65%)	DNI is associated with gingivitis and P
**Lymphocyte to monocyte ratio (LMR)**	Mishra et al., 2022, India^138^	Automated hematology analyzer (Sysmex XN ‐ 1000)	Case–control	*n* = 148 (80 male, 68 females, and mean age 31 years)	108 generalized stage III grade C P^52^ versus 40 H	Yes	Only nonsmokers	−	−	LMR of P: 7.26% (SD = ±4.94%), LMR of H: 9.31% (SD = ±4.88%), significant difference between H and P patients (*p* = 0.004); LMR is significant negative associated with mean PPD, mean CAL, and not associated with mean BOP, mean mGI, and mean plaque index; ROC analysis yielded cutoff values of ≤7.16% in predicting risk of P, predictive validity as indicated by AUC = 0.654 (CI 0.529–0.691, *p* = 0.003); Based on these cutoff values, OR of *p* = 4.93 (CI 2.26–10.76, *p* = 0.0001) with each 0.1 decrease in LMR; Logistic regression analysis (age, male gender, and BMI as explanatory variables and LMR values as dependent variables): age is a significant predictor of differences (*p* = 0.004), not male gender (*p* = 0.36) and BMI (*p* = 0.58); LMR: sensitivity = 70.37% (CI 60.81% ‐ 78.77%), specificity = 67.50% (CI 50.87% ‐ 81.42%), and diagnostic accuracy = 69.59% (CI 61.50%–76.88%)	LMR is associated with P
**Platelet to lymphocyte ratio (PLR)**	Acharya et al., 2019, India^90^	Automated hematology analyzer (Sysmex pocH‐100i;)	Case–control and prospective cohort	*n* = 60 (30 males, 30 females), mean age P 45.08 years and H 39.6 years	30 CP^130^ versus 30 H	No	Not reported	Not reported	Not reported	PLR of pretreatment with CP: 121.08 (SD = ±43.58), PLR of post‐treatment: 80.0 (SD = ±26.50), *p* < 0.0001, PLR of H: 111.6 (SD = 37.36); PLR in pre‐ and post‐treated patients was not associated to local periodontal parameters; cutoff point by ROC for pre‐ versus post‐treated patients = 80.205 (sensitivity = 0.867, specificity = 0.622, and AUC = 80.6%)	Significant difference of PLR between pre‐ and post‐treated CP patients
	Lu et al., 2021, China^91^	Automated hematology analyzer (Sysmex XS ‐1000)	Case–control	*n* = 505 (205 males, 300 females, mean age 27 years)	372 generalized AgP^136^ versus 133 H	Yes	Only nonsmokers	−	−	PLR of AgP: 132.23 (SD = ±45.48), PLR of H: 125.82 (SD = ±42.41), no significant difference between H and AgP (*p* = 0.157), AgP group: PLR was not associated to PPD, CAL, and BOP	PLR is not associated with generalized AgP
	Mishra et al., 2022, India^137^	Automated hematology analyzer (Sysmex XN ‐ 1000)	Case–control	*n* = 630 (324 males, 306 females, mean age 34 years)	315 generalized stage III grade C P^52^ versus 315 H^134^	Yes	Only nonsmokers	−	−	PLR of P: 135.58 (IQR = 112.73–164.08), PLR of H: 124.16 (IQR = 102.14–154.09), significant difference between H and P (*p* = 0.02); ROC cutoff value >126.08, MLRA: high significant association of P with PLR OR = 2.16 (CI 1.56–3.01, *p* < 0.0001) after adjusting for BMI, WBC count, and oral hygiene habits; predictive validity in discriminating P patients from H individuals as depicted by AUC = 0.788 (CI 0.754–0.819, *p* < 0.0001); PLR: sensitivity = 64.12% (CI 58.55–69.42%), specificity = 53.33% (CI 47.65–58.95%), and diagnostic accuracy = 58.73% (CI 54.77–64.03%)	PLR is associated with P
	Mishra et al., 2022, India^138^	Automated hematology analyzer (Sysmex XN ‐ 1000)	Case–control	*n* = 148 (80 male, 68 females, mean age 31 years)	108 generalized stage III grade C P^52^ versus 40 H	Yes	Only nonsmokers	−	−	PLR of P: 143.98 (SD = ±59.0), PLR of H: 134.16 (SD = ±38.53), no significant difference between H and P patients (*p* = 0.574); PLR was significant positive associated with mean PPD, mean plaque index, and not associated with mean CAL, mean BOP, and mean mGI	PLR is not associated with P
	Torrungruang et al., 2018, Thailand^141^	Automated hematology analyzer (Cell‐Dyn Ruby System; Abbott Diagnostics)	Cross‐sectional	*n* = 2036 (gender and age not reported)	1555 normoglycemic, 331 IFG, and 150 diabetes patients^142^; divided into: 365 no/mild P, 1058 moderate P, and 613 severe P^143^	Yes	Nonsmokers, former smokers, and current smokers with significant differences	+	+	PLR of normoglycemic: 120.7 (IQR = 97.3–149.3), PLR of IFG: 109.6 (IQR = 91.2–135.2), PLR of diabetes: 103.0 (IQR = 81.7–127.6), significant differences between groups (*p* < 0.001); PLR of no/mild P: 125.2 (IQR = 101.1–156.7), PLR of moderate P: 118.8 (IQR = 96.5–145.9), PLR of severe P: 109.8 (IQR = 87.0–136.4), comparison between no/mild/moderate P versus severe P was more likely to have lower PLR (*p* = 0.015); after controlling for P severity, PLR was negatively associated with diabetes (*p* = 0.007)	PLR is highly associated to patients with/without diabetes and with/without P
**Platelet distribution width (PDW)**	Mutthineni et al., 2021, India^144^	Automated cell counter (UBM F‐19)	Cross‐sectional	*n* = 75 (gender not reported, age between 35 years and 50 years)	Severe CP^143^ versus moderate CP versus H	Yes	Only nonsmokers	Not reported	Not reported	PDW of H: 10.51 (SD = ±2.71), PDW of moderate CP: 10.61 (SD = ±1.58), PDW of severe CP: 10.87 (SD = ±1.40), *p* = 0.805 (ANOVA with post hoc Games‐Howell test), mean PDW levels showed no significant changes from normal to diseased individuals; one‐sample t‐test showed significant differences between groups (*p* < 0.001)	Association unclear
	Temelli et al., 2018, Turkey^139^	Selective coronary angiography with contrast agent by means of Judkins technique in multiple projections; clinical chemistry analyzer (AU5800; Beckmann Coulter)	Case–control	*n* = 77 (47 males, 30 females, and mean age Group 1 = 59.5 years, Group 2 = 57.5 years, Group 3 = 50.0 years, Group 4 = 49.0 years)	41 P^136^ versus 36 non‐P (gingivitis, as described in the discussion^140^); divided into group 1 = CAD with P (*n* = 20), group 2 = CAD without P (*n* = 20), group 3 = non‐CAD with P (*n* = 21), and group 4 = non‐CAD without P (*n* = 16)	Yes, of periodontists and cardiologists	Discussed smoking and found no significant differences	+	−	PDW of group 1: 17 (min. = 15.7, max. = 18.3), PDW of group 2: 16.35 (min. = 15.7, max. = 17.4), PDW of group 3: 16.6 (min. = 15.9, max. = 17.3), and PDW of group 4 = 16.45 (min. = 15.9, max. = 16.9), significant differences among the groups with CAD and P versus non‐CAD and non‐P, with CAD and with P versus with CAD and non‐P and with CAD and with P versus non‐CAD with P (*p* < 0.01); significant association between PDW and CAL (*p* = 0.033)	PDW is associated to CAD patients with/without P
**Plateletcrit (PCT)**	Mutthineni et al., 2021, India^144^	Automated cell counter (UBM F‐19)	Cross‐sectional	*n* = 75 (gender not reported, age between 35 years and 50 years)	Severe CP^143^ versus Moderate CP versus H (*n* not reported)	Yes	Only nonsmokers	Not reported	Not reported	PCT of H: 0.19 (SD = ±0.04), PCT of moderate CP: 0.30 (SD = ±0.06), PCT of severe CP: 0.42 (SD = ±0.09), and mean PCT levels showed significant changes from normal to diseased individuals (ANOVA with post hoc Games‐Howell test, *p* < 0.001); One‐sample t‐test showed for H and severe P significant differences (*p* < 0.001) and for moderate P, no significant differences (*p* = 0.132)	PCT is highly associated with P
	Ustaoglu et al., 2020, Turkey^145^	Automated hematology analyzer (Cell‐Dyn 3700 System; Abbott Diagnostics)	Case–control	*n* = 114 (55 males, 59 females, mean age P group 37.4 years and H group 35.6 years)	57 stage III P^52^ versus 57 H	Yes	Only nonsmokers	−	−	PCT of P: 0.223 (SD = ±0.04), PCT of H: 0.196 (SD = ±0.04), *p* < 0.001; significant associations between PCT and mean PPD, mean CAL, and BOP (all *p* < 0.001)	PCT is highly associated with P
**Systemic immune inflammation index (SII)**	Cao et al., 2023, USA^149^	Automated hematology analyzer (company not reported)	Cross‐sectional	*n* = 10 301 (data from National Health and Nutrition Examination Survey, 48.86% male, 51.14% female, mean age 50.88 years)	62.29% moderate/severe P^143^ versus 37.71% no/mild P	Not reported	Nonsmokers, former smokers, and smokers; significant differences	Not reported	Not reported	Association between SII and p followed a j‐shape curve (*p* for nonlinearity <0.001); Risk of moderate/severe P decreased by 17% per unit SII when log_2_(SII) ≤8.66 (or = 0.83; CI = 0.69–0.999) and increased by 19% per unit when log_2_(SII) >8.66 (or = 1.19; ci 1.02–1.38); Significant differences between SII levels: average age, gender, ethnicity, marital status, smoking habit, obesity rates, diabetes prevalence, hypertension rates, mean cal, mean ppd, number of sites ppd ≥4 mm, number of sites cal ≥3 or 5 mm, and the prevalence of P	SII is highly associated with P
	Mishra et al., 2022, India^137^	Automated hematology analyzer (Sysmex XN ‐ 1000)	Case–control	*n* = 630 (324 males, 306 females, mean age 34 years)	315 generalized stage III grade C P^52^ versus 315 H^134^	Yes	Only nonsmokers	−	−	SII of P: 723.87 (IQR = 605.16–968.37), SII of H: 537,74 (IQR = 468.38–588.41) (×10^9^/L), significant difference between H and P patients (*p* < 0.0001); ROC cutoff value >591.48, MLRA: high significant association of P with SII (OR = 11.86, CI 7.99–17.59, *p* < 0.0001) after adjusting for BMI, WBC count, and oral hygiene habits; predictive validity in discriminating P patients from H individuals as depicted by AUC = 0.766 (CI 0.731–0.799, *p* < 0.0001); SII: sensitivity = 81.27% (CI 76.51–85.42%), specificity = 76.50% (CI 71.43–81.08%), and diagnostic accuracy = 78.89% (CI 75.49–82.01%)	SII is highly associated with P
**Red blood cell distribution width (RDW)**	Anand et al., 2014, India^146^	Automated hematology analyzer (BC‐3000 Plus; Shenzhen Mindray Bio‐Medical Electronics)	Case–control	*n* = 122 (65 males, 57 females, mean age P group 32.8 years and H group 30.4 years)	64 generalized AgP^136^ versus 58 H	Yes	Nonsmokers, former smokers, and current smokers with significant differences (logistic regression model)	−	−	RDW of AgP: 15.27% (SD = ±1.44%), RDW of H: 15.16% (SD = ±1.07%), no significant differences between groups (*p* = 0.617)	RDW is not associated with generalized AgP
	Bhattacharya et al., 2022, India^131^	Not reported	Cross‐sectional	*n* = 80 (40 males, 40 females, mean age P 41.8 years and H 36.93 years)	40 P^132^ versus 40 H	Yes	Only nonsmokers	+	−	RDW of P: 50.31% (SD = ±8.93%), RDW of H: 40.52% (SD = ±5.48%), significant differences between groups (*p* < 0.001)	RDW is highly associated with P
	López et al., 2012, Chile^147^	Automated hematology analyzer (Cell‐Dyn 3500 System; Abbott Diagnostics)	Case–control	*n* = 160 (72 males, 88 females, mean age P 16.7 years and H 16.4 years)	87 P versus 73 H	Yes	Nonsmokers and smokers; no significant difference	−	+	RDW of P: 14.40% (SD = ±0.87%), RDW of H: 14.38% (SD = ±0.83%), no significant difference between groups (*p* = 0.887)	RDW is not associated with P
	Sridharan et al., 2021, India^148^	Automated hematology analyzer (Sysmex KX ‐ 21 *N*)	Case–control	*n* = 80 (26 males, 54 females, and mean age Group 1 = 41.6 years, Group 2 = 50.8 years, Group 3 = 42.6 years, and Group 4 = 48.4 years)	Group 1: non hypertensive without P (*n* = 20), group 2: nonhypertensive with P (*n* = 20), group 3: hypertensive without P (*n* = 20), and group 4: hypertensive with P (*n* = 20)	Yes	Nonsmokers, former smokers, and smokers; no significant differences	+	−	P groups (hypertensive and nonhypertensive) showed significant association with RDW (*p* < 0.001); mean PI, GI, PPD, CAL (dependent variables), and RDW were significant associated with P (hypertensive and nonhypertensive, *p* < 0.001)	RDW is highly associated with P
	Temelli et al., 2018, Turkey^139^	Selective coronary angiography with contrast agent by means of Judkins technique in multiple projections; clinical chemistry analyzer (AU5800; Beckmann Coulter)	Case–control	*n* = 77 (47 males, 30 females, and mean age Group 1 = 59.5 years, Group 2 = 57.5 years, Group 3 = 50 years, and Group 4 = 49 years)	41 P^136^ versus 36 non‐P (gingivitis^140^); divided into group 1 = CAD with P (*n* = 20), group 2 = CAD without P (*n* = 20), group 3 = non‐CAD with P (*n* = 21), and group 4 = non‐CAD without P (*n* = 16)	Yes, of periodontists and cardiologists	Discussed smoking and found no significant differences	+	−	RDW of group 1: 14% (min. = 12.3%, max. = 17.2%), RDW of group 2: 14.1% (min. = 12.8%, max. = 16.3%), RDW of group 3: 13.6% (min. = 12.3%, max. = 20.3%), And RDW of group 4: 13% (min. = 12.3%, max. = 16.6%), no association between groups; moderate association between PPD and RDW (*p* = 0.049) in non‐CAD groups with/without P	RDW is not associated to patients with/without CAD and/or P
	Ustaoglu et al., 2020, Turkey^145^	Automated hematology analyzer (Cell‐Dyn 3700 System; Abbott Diagnostics)	Case–control	*n* = 114 (55 males, 59 females, and mean age P group 37.4 years and H group 35.6 years)	57 stage III P^52^ versus 57 H	Yes	Only nonsmokers	−	−	RDW of P: 14.80% (SD = ±1.88%), RDW of H: 15.20% (SD = ±1.51%), no significant difference between H and P (*p* = 0.212); no associations between PCT and mean PPD, mean CAL, and BOP	RDW is not associated with P

Abbreviations: %, percentage; −, no significant differences between groups; +, significant differences between groups; AgP, aggressive periodontitis; AUC, area under the curve; BMI, body mass index; BOP, bleeding on probing; CAD, coronary artery disease; CAL, clinical attachment level; CI, 95% confidence interval; SD, mean standard deviation; CP, chronic periodontitis; DNI, delta neutrophil index; H, systemically and periodontal ealthy controls; IFG, impaired fasting glucose; IQR, interquartile range; LMR, lymphocyte to onocyte ratio; mGI, modified gingival index; MLRA, multiple logistic regression analysis; *n*, umber; NLR, neutrophil to lymphocyte ratio; OR, odds ratio; P, periodontitis; PCT, plateletcrit; PDW, platelet distribution width; PLR, platelet to lymphocyte ratio; PPD, probing pocket depth; RDW, red blood cell distribution width; ROC, receiver operating characteristics; SII, systemic immune inflammation index; WBC, white blood cell.

The included studies not only tried to find a possible association between the systemic blood indices and periodontitis, but also partly analyzed an association to systemic diseases like cancer. These in turn are often being associated with periodontal diseases. Therefore, these associations between the diseases are also briefly explained. When evaluating cancer in the periodontitis patients, it should be noted that at least 50% of all patients suffer comorbidity. Periodontitis patients are often “sick” patients.^50^, ^150^

In clinical practice, the NLR is the most widely used index and is associated to periodontitis and cancer.

## 4 NEUTROPHIL TO LYMPHOCYTE RATIO

### 4.1 The basis of NLR

**FIGURE 2:**
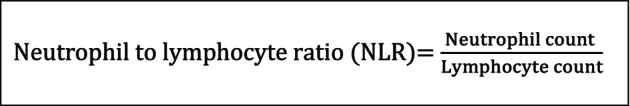
Calculation of the neutrophil to lymphocyte ratio (NLR).

The NLR is a marker derived from a standard full blood cell count test. It is calculated by dividing the neutrophil count by the lymphocyte count (both per microliter of blood, Figure 2). NLR is an effective predictor than using either measurement neutrophil or lymphocyte counts alone since it includes both parameters instead of one.^91^, ^151^ The mean NLR in healthy Caucasian populations (Non‐Hispanic White without periodontal investigation) is 2.24.^152^ This value is comparatively higher than those observed in other ethnical groups with periodontal examination, for example, 1.65 in healthy South Koreans,^153^ 1.76 in healthy Non‐Hispanics of African lineage,^152^ 1.84 in healthy Han Chinese,^91^ and 1.86 in healthy Asian Indians.^90^ Given the demonstrated racial differences in immune responses,^154^ it is plausible that the average NLR in healthy individuals exhibits racial predisposition.^152^ This ratio is increasingly recognized for its prognostic and diagnostic value in various clinical contexts, particularly in assessing systemic inflammation, infection, and predicting outcomes of various diseases and shows an association to inflammatory markers such as CRP.^155^ A high NLR can indicate a relative increase in neutrophils and/or a decrease in lymphocytes. This change often suggests an active inflammatory process, as neutrophils are typically elevated in acute and chronic infections. Lymphocyte counts increase during a viral infection and decreases in infections after trauma or when human immunodeficiency virus spreads. In clinical practice, NLR is used as a marker for systemic inflammation. NLR is advantageous due to its simplicity, cost‐effectiveness, and accessibility, as it can be calculated from routine blood tests. However, its interpretation should be integrated with other clinical findings and laboratory tests for a comprehensive evaluation of a disease.

An elevated NLR in cardiovascular diseases, particularly heart failure and myocardial infarction, is associated with a poor prognosis, with NLR values often exceeding 3.^25^, ^26^, ^27^ For instance, a recent systematic review by Angkananard et al.^28^ on NLR and cardiovascular disease risk, identifying cutoff values ranging from 1.80 to 2.60 for coronary artery disease (CAD), 2.19 to 5.70 for acute coronary syndrome, and 3.0 to 3.17 for cerebrovascular stroke. Furthermore, a higher NLR has been linked to poor glycemic control and an increased risk of type 2 diabetes mellitus, with varying cutoff values from 2.44 to 4.34.^29^, ^30^, ^31^ In diabetic patients, who often have comorbid systemic conditions, NLR values can exceed 4.^32^ An NLR >3 has been associated with increased 2‐year follow‐up mortality in medical in‐patients with multiple chronic conditions.^156^ Additionally, NLR has been recognized as a significant prognostic marker for people with obstructive sleep apnea with mean NLR values ranging from 1.61 to 4.18,^157^ deteriorating renal function^158^ and lung disorders.^34^

### 4.2 The use of NLR in cancer

NLR has emerged as a widely studied and recognized biomarker in the field of cancer. To date, over 300 reviews have been published exactly on this topic. The underlying mechanism involves the role of neutrophils in promoting tumor‐associated inflammation, angiogenesis, and immune suppression, while decreased lymphocyte counts contribute to compromised antitumor immune responses. As described in a systematic review and meta‐analysis by Cupp et al.,^159^ numerous studies across diverse malignancies have reported a consistent association between elevated NLR and advanced disease stages, tumor aggressiveness, and reduced overall survival. In an additional meta‐analysis, Templeton et al.^48^ illustrated that a NLR >4 is independently associated with a reduced overall survival in solid tumors. Moreover, NLR serves as a predictive factor for cancer‐specific survival, progression‐free survival, and disease‐free survival, showing highly significant hazard ratios of more than 1.61 (95% CI = 1.36–1.91), 1.63 (95% CI = 1.39–1.91), and 2.27 (95% CI = 1.85–2.79), respectively. However, while NLR holds promise as a prognostic marker, its universal applicability necessitates careful consideration of cancer type‐specific variations and a precise cutoff value for every type of cancer has not been decided so far.

### 4.3 Findings from the use of NLR in periodontology

In patients with periodontitis and concurrent systemic diseases, NLR values may reflect both periodontitis and systemic conditions. This requires consideration of potential confounding systemic diseases influencing NLR. These observations suggest that NLR could be a linking factor between periodontitis and other systemic inflammatory diseases.

NLR is the most studied “cancer” biomarker in periodontitis. Many systemic blood indices others than NLR have been studied. Six of nine studies did find an association between NLR and periodontitis. However, the largest study with 2036 participants included diabetic patients,^141^ which is a confounding factor. While previous research has documented that high levels of NLR are associated with type 2 diabetes,^31^, ^160^, ^161^ the specific association between these altered NLR levels and periodontitis has only been reported in one study by Torrungruang et al.^141^ This cross‐sectional study investigated the relationship between different glycemic status, NLR, and periodontitis in a Thai population. The study benefited from its robust sample size, encompassing 2036 subjects, which facilitated exhaustive control for potential confounding factors. Based on the 2014 diagnostic criteria by the American Diabetes Association,^142^ the study participants were divided into three groups: normoglycemia (*n* = 1555), impaired fasting glucose (*n* = 331), and diabetes (*n* = 150, without differentiation whether type 1 or type 2). Analysis of the parts of NLR showed that counts of both neutrophils and lymphocytes were significantly elevated with worsening glycemic status and with increasing severity of periodontitis. Similar outcomes were observed when utilizing mean pocket probing depth (PPD) or clinical attachment level (CAL) as indicators of periodontitis severity. Mean PPD and CAL values were higher among the diabetic cohort, subsequently lower by the impaired fasting glucose and normoglycemic groups. As a result, the absence of a link between NLR and diabetes within this study might be explained by simultaneous elevations in both neutrophil and lymphocyte counts with worsening of the glycemic status. Nonetheless, in the group with severe periodontitis, these increases appeared more distinct for neutrophils. One weakness of this study is its high heterogeneity of the study population with significant differences in age, gender, and smoking habits.

The second study which included patients with type 2 diabetes mellitus and other systemic diseases confirmed the results of Torrungruang et al.^141^ Thus, these systemic risk factors were investigated in a study by Doğan et al.^135^ across a cohort of 180 patients with and without periodontitis. The study had some limitations, such as no specified periodontal classification, a lack of diet analysis, participants had poor oral hygiene habits, and included smokers and nonsmokers. As a result of this study, periodontitis patients with type 2 diabetes (*n* = 75), hyperlipidemia (*n* = 99), obese (*n* = 119), and menopause (*n* = 37) compared to healthy controls (*n* = 28) showed no significant difference in NLR. Only the hyperlipidemia group (*n* = 99) showed a significant higher NLR (2.56 ± 1.50) in the periodontitis group compared to the nonperiodontitis group (NLR: 2.05 ± 1.31) and a significant positive association between clinical periodontal parameters and NLR was reported. The association between periodontitis and hyperlipidemic patients had already been reported in other studies.^162^, ^163^ The data cumulatively indicate that hyperlipidemia may increase the inflammatory processes associated with periodontitis. Specifically, hypercholesterolemia contributes to monocytic activity, which in turn could increase neutrophil count.^164^

Another study that included patients with CAD confirmed the results of Torrungruang et al.^141^ and Doğan et al.^135^ Temelli et al.^139^ evaluated in a cross‐sectional design the relationship between NLR in patients with and without CAD, respectively, and also with and without periodontitis. A total of 77 patients who underwent coronary angiography due to suspected CAD and periodontal assessment, were enrolled. The NLR was not significantly different among the study groups with and without CAD and periodontitis, similar to the neutrophil and leukocyte counts.

The absence of significant differences of the hematological parameters and NLR may be attributable either to the acute severity of periodontitis within the study participants or the limited sample size encompassed by the investigation.

Çetin Özdemir et al.^133^ investigated the potential association of NLR in relation to three different groups in a Turkish systemically healthy nonsmoking population: periodontally healthy (*n* = 26), gingivitis (*n* = 26), and generalized stage III periodontitis (*n* = 26).^52^, ^134^ The results demonstrated no significant differences in NLR, neutrophil counts, and lymphocyte counts between the groups.

Five out of six studies who excluded patients with systemic diseases demonstrated a significant association between NLR and periodontitis. Mishra et al.^137^ conducted one of the largest studies including 630 individuals in a multicenter design. They included 315 generalized stage III grade C periodontitis patients and 315 periodontally and systemic healthy individuals. Participants in this study were age and gender matched. Additionally, smokers were excluded.^165^ The study identified a notably elevated body mass index (BMI) in subjects with periodontitis. Although the BMI values of numerous participants fell within the standard range, there was a moderate statistically higher BMI in the periodontitis group in comparison to the healthy group. Several studies observed an association between BMI and reduced total WBC count and increased counts of neutrophils and lymphocytes in GAgP patients.^166^, ^167^ Furthermore, the results of selected studies indicated that an increased BMI is linked to an increase in NLR.^91^, ^168^ While absolute neutrophil counts (5.14 vs 4.6) were significantly higher in the periodontitis group, absolute lymphocyte counts (1.93 vs 2.2) were significantly lower. This resulted in a significantly different NLR of 2.74 in patients with periodontitis compared to 2.13 in healthy individuals. Receiver operating characteristics (ROC) yielded cutoff value of >2.44 in discriminating patients with periodontitis. Based on this cutoff value, multiple logistic regression analysis indicated significant association of periodontitis with NLR (OR = 9.65, 95% CI = 6.59–14.13) after adjusting for oral hygiene habits, BMI, and WBC count. The sensitivity of NLR was 74%, the specificity was 80%, and diagnostic accuracy was 77%.

Mishra et al.^138^ also investigated the association between severe periodontitis in patients in India between 20 and 40 years. Healthy controls were assessed in a separate retrospective and single center study where the participants were age, gender, and BMI matched. Furthermore, a multicenter study was performed by the same group.^137^ The major findings revealed significantly higher neutrophil counts and NLR (2.84 vs 2.10) values in periodontitis patients. Also, the risk of severe grade C periodontitis increased with the height of NLR values. The NLR was significantly positive related with all recorded periodontal parameters like CAL, PPD, and bleeding on probing (BOP). The ROC analysis yielded a cutoff value of >2.15. This cutoff value determined an OR of 11.4 for severe grade C periodontitis. Every 0.1 increase in NLR stands for a major elevation of the risk to have periodontitis. This leads to a very high sensitivity of 77%, a specificity of 78%, and a total diagnostic accuracy of 77% in predicting severe grade C periodontitis in in young adults in an Indian population. These cutoff values identified by Mishra et al.^138^ diverged from those established in a study on young Chinese adults. Additionally, the predictive accuracy of the leukocyte ratios was observed to be superior in opposite to Lu et al.^91^ This variation in cutoffs underscores the necessity for further investigations across diverse racial populations.

As discussed previously in this review, this underscores the significance of systemic inflammation as a critical factor in determining the severity of GAgP.^169^, ^170^ Neutrophils and lymphocytes serve as pivotal components in both inflammatory and immunological reactions observed in patients diagnosed with GAgP.^171^, ^172^ Studies highlighted a significant increase in the number of neutrophils and a significantly lower numbers of lymphocytes in the periphery blood of GAgP patients. This suggests that clinical periodontal parameters might be directly associated to the numbers of these cell types.^172^ The only study including GAgP patients based on the old periodontitis classification^136^ was published by Lu et al.^91^ The group by Mishra et al.^137^, ^138^ included comparable grade C periodontitis^52^ patients. Notably, the BMI of the 372 Chinese GAgP patients was significant higher (21.4 vs 22.2 kg/m^2^) than in the 133 healthy controls. No significant differences were noted in the distribution of age and gender between the two groups. Moreover, a statistically significant difference was observed in neutrophil numbers, while lymphocyte counts displayed no evident discrepancies. NLR was significantly higher in GAgP patients than in the control group (mean NLR = 2.34 vs mean NLR = 1.84). Subsequently, the values of NLR were differentiated in different subgroups. The distribution in the NLR subgroups differed between the GAgP and healthy groups. Significantly, more patients of the GAgP group were found in the NLR 2–3 and NLR ≥3 subgroups. Additionally, the NLR values were positively associated with PPD and CAL in patients with GAgP. Furthermore, an incremental rise of one unit in the NLR corresponded to respective increases of 0.41 mm in PPD and 0.57 mm in CAL. Additionally, the cutoff point was defined by 1.92. Lymphocyte counts exhibited a negative association with CAL, while neutrophil counts were significantly linked to different periodontal parameters such as CAL and PPD. Lu et al. (2021) concluded that NLR could serve as a prospective biomarker for inflammation and disease severity in Chinese GAgP patients.

One study analyzing NLR before and after an anti‐infective therapy was published by Acharya et al.^90^ 30 Indian chronic periodontitis patients were provided oral hygiene instructions at baseline. SRP was divided into two appointments 1‐week apart with additionally oral hygiene instructions. After 1 month, the PPD and the CAL showed a reduction of 1 mm to 5.83 mm and 1.25 mm to 6.51 mm which still indicates severe periodontitis. The limited reduction of the plaque index (1.86 vs 0.97) and gingival index (1.81 vs 1.24) did not indicate a successful periodontal treatment. Nevertheless, NLR showed a significant reduction from 1.90 to 1.48. This determined a cutoff point of 1.54 between pre‐ and post‐treated patients. The NLR was positively associated to PPD and CAL before periodontal treatment and negatively after treatment, which means that the therapeutic intervention had a remarkable influence on NLR. It has to be considered that the selection of the controls was disputable and the smoking status of the patients was not addressed. The 30 periodontal and systemic healthy controls included in this study showed a mean NLR of 1.86, which was close to the pretreatment values in the periodontitis group. Furthermore, a comparison between healthy controls and patients before treatment was not reported.

A recent meta‐analysis included some of the above referred studies.^90^, ^91^, ^133^, ^135^, ^138^, ^139^, ^141^ It was found that the mean NLR was statistically significant higher by 0.41, in the periodontitis group compared to the control group.^173^ These findings indicate that the increased NLR is related to periodontitis and may be an important systemic blood index. Depending on racial predisposition, it can be hypothesized, in cases with NLR >2, that periodontitis has systemic effects.

## 5 DELTA NEUTROPHIL INDEX

### 5.1 The basis of DNI

**FIGURE 3:**
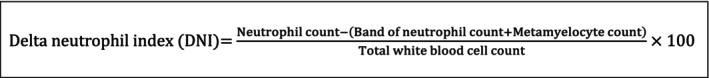
Calculation of the delta neutrophil index (DNI).

The DNI is a hematological parameter used primarily to assess the severity and prognosis of infections, particularly in the context of systemic inflammatory response syndrome (exaggerated defense response of the immune system to a noxious stressor resulting in dysregulation of the immune response) and sepsis. It quantifies the distribution of immature to mature neutrophils in the blood. DNI can be measured by deducting the proportion of mature polymorphonuclear leukocytes from the aggregate of myeloperoxidase‐reactive cells.^174^ As a result of the emergence of immature granulocytes, changes occur in alterations in peripheral total WBC counts during the granular leukocyte differentiation process.^174^, ^175^ This is indicative of an ongoing inflammatory process or infection. The DNI is calculated using automated blood analyzers that measure the difference in the absorbance of light by mature and immature neutrophils. These analyzers employ two different wavelengths to differentiate between these cell types based on their nuclear segmentation and cytoplasmic granularity. Figure 3 illustrates how DNI is calculated. The DNI provides a more precise measure of the proportion of immature to mature neutrophils than a simple immature granulocyte count, thereby offering a more accurate reflection of the body's response to (severe) infection or inflammation.

The DNI is characterized by a half‐life of approximately 3 h, substantially shorter than the 24–30 h half‐life of procalcitonin, also used as a marker for bacterial infectious diseases.^176^ This shorter half‐life allows for a more rapid reflection of a patient's status of infection, proving advantageous in monitoring and evaluating the effectiveness of therapeutic interventions. Generally, a higher DNI value correlates with increased systemic inflammation. The calculation and reporting of DNI are straightforward and does not incur additional costs.^133^, ^177^ Previous studies highlight the benefits of DNI as a diagnostic and prognostic marker in various infectious or inflammatory diseases such as septic shock, bacteremia,^178^ sudden cardiac arrest,^179^ and covid‐19.^180^

### 5.2 The use of DNI in cancer

The DNI has been investigated as a potential biomarker in the context of cancer, reflecting alterations in neutrophil subpopulations and their response to inflammatory stimuli. In various malignancies, elevated DNI levels have been observed, suggesting a potential association with the inflammatory processes associated with tumor development and progression. The heightened inflammatory state in cancer often leads to an increased release of immature neutrophils, contributing to elevated DNI. Studies have reported that an elevated DNI is associated with adverse prognostic outcomes in cancer patients. Ko et al.^181^ investigated the DNI in early diagnosed suspected acute promyelocytic leukemia patients and found DNIs between 52% and 56% at different time periods during treatment. In nonacute promyelocytic leukemia, DNIs varied between 10% and 12%. Increased predictability for acute promyelocytic leukemia was associated with a DNI >24.2% in early diagnosed patients. Study groups from a university clinic in Turkey published studies on thyroid malignancy,^182^ axillary metastasis of breast cancer,^183^ and renal cell carcinoma patients.^184^ In all studies, pretreatment DNI was significantly higher in the malignant group compare to benign or nonmalignant group with high sensitivity and specificity in detecting cancer. After treatment, the differences were negligible. No systemic reviews or meta‐analyses have been published yet. Therefore, it is too early to assess whether the DNI is a useful marker for the detection of carcinoma.

### 5.3 Findings from the use of DNI in periodontology

So far, only one study by Çetin Özdemir et al.^133^ has investigated the association between periodontitis and DNI. As described above, the study also investigated the relationship between periodontitis and DNI in three different groups in a Turkish population: periodontally healthy, gingivitis, and generalized stage III periodontitis patients.^52^, ^134^ The DNI was significantly higher in the periodontitis and gingivitis group compared to the health group. Furthermore, the DNI demonstrated a positive association to all periodontal parameters. The ROC analysis revealed that DNI exhibited a sensitivity of 91% and specificity of 65% with an optimal cutoff at 0.25 in diagnosing periodontal disease, suggesting its potential as a biomarker for periodontitis. But DNI has shown promise as a diagnostic tool for infection; its role in chronic inflammatory diseases like periodontitis remains underexplored. The shorter half‐life of DNI predicts more effectively systemic inflammatory load and may serve as a better monitor marker for therapeutic efficacy. Therefore, DNI could be a potential novel biomarker for periodontal diseases. However, further studies are needed to verify the results.

## 6 LYMPHOCYTE TO MONOCYTE RATIO

### 6.1 The basis of LMR

**FIGURE 4:**
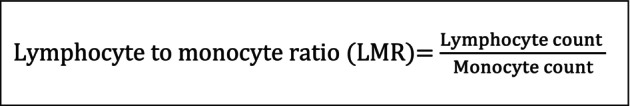
Calculation of the lymphocyte to monocyte ratio (LMR).

LMR operates as an inflammatory complex analogous to the role of NLR.^55^ This marker presents a distinctive systemic inflammatory indication, integrating two distinct metrics of inflammation (Figure 4). A limited number of studies have determined reference values of LMR in healthy adults, for example, in South Korea (5.31)^153^ and in China (5.3).^185^ However, the values are influenced by ethnicity, age, and gender.^138^, ^153^, ^185^, ^186^ Recent studies indicated a significant reduction in LMR levels among rheumatoid arthritis patients from 6.1 (healthy) to 4.04 (patients).^186^ Moreover, an investigation assessing the relationship between LMR and the severity of CAD determined that severe atherosclerosis is associated with an LMR value of <5.06.^55^

### 6.2 The use of LMR in cancer

LMR has also emerged as a prognostic marker in cancer research, reflecting the complex interplay between the immune system and tumorigenesis. Elevated LMR has been associated with better prognosis in various malignancies, suggesting a potential role as a favorable prognostic indicator. The LMR's predictive value is attributed to the dynamic interaction between lymphocytes, key effectors of antitumor immunity and monocytes, which can exhibit pro‐tumorigenic or antitumorigenic activities. High LMR is generally indicative of a robust immune response, suppressing tumor progression and enhancing the overall anticancer defense mechanisms.^187^, ^188^ Two meta‐analyses showed an association between high pretreatment LMR levels and improved overall survival and disease‐free survival for pancreatic cancer (HR = 0.68, 95% CI = 0.58–0.80; HR = 0.55, 95% CI = 0.31–0.96)^187^ and colorectal cancer (HR = 0.57, 95% CI = 0.52–0.62; HR = 0.77, 95% CI = 0.70–0.84).^188^ Conversely, a reduced LMR value may signify compromised immune system, allowing tumor manifestation and progression. Numerous meta‐analyses across different types of cancer reported an inverse association between LMR and worse overall survival, for example, glioma (HR = 1.35, 95% CI = 1.13–1.61),^189^ lung cancer (HR = 1.61, 95% CI = 1.45–1.79),^190^ esophageal squamous cell carcinoma (HR = 0.67, 95% CI = 0.58–0.78),^191^ gastric cancer (HR = 0.66, 95% CI = 0.54–0.82),^192^ and rectal cancer (HR = 1.57, 95% CI = 1.29–1.90).^193^ Finally, a low LMR, but not a high LMR, was often inversely correlated with complete pathologic remission rate (complete absence of cancer cells in tissue after treatment).^189^, ^190^, ^191^, ^192^, ^193^ A review by Gu et al.^194^ provides a useful overview. However, the cited meta‐analyses were more recently published. Pretreatment LMR cutoff values between 2.8 and 4.6 indicate high heterogeneity between studies, patients, and types of cancer.^187^, ^189^, ^192^, ^195^ Therefore, there is no generalized cutoff value.

### 6.3 Findings from the use of LMR in periodontology

The correlation between LMR and periodontitis remains largely unexplored, but such a relationship could provide valuable insights into systemic inflammation and contribute to the diagnosis and prognostic evaluation of periodontitis. Only one study by the group of Mishra et al.^138^ investigated LMR in periodontitis patients and healthy controls. There were no significant differences in age and gender distribution between the groups. The major outcome of the study revealed a significant lower LMR values (7.26 vs 9.31) in patients diagnosed with generalized stage III grade C periodontitis. Furthermore, LMR is significantly negative related with PPD and CAL. ROC analysis yielded cutoff values of ≤7.16 in predicting the risk of periodontitis. Based on these cutoff values, the OR of having stage III grade C periodontitis was 4.9327 with each 0.1 decrease in LMR. The predictive validity of LMR, as indicated by the area under the curve, was moderately valid by 0.654. Logistic regression analysis demonstrated that age but not gender and BMI is a significant predictor of differences in LMR. Finally, this study highlights that the diagnostic accuracy of LMR was 70% with a sensitivity of 70% and a specificity of 68%. A limited number of studies have explored the reference values for LMR as described above, with findings indicating cutoff values lower than those reported in the study by Mishra et al.^138^ These cutoff values in young Indian periodontitis patients^138^ were different from the study accomplished on Chinese young adults and the predictive validity of the leukocyte ratios was also higher than in a Chinese population.^91^

The variation in cutoff values indicates the need for expanded research across different ethnical groups. Consequently, the reduced LMR observed in periodontitis patients, as reported by Mishra et al.,^138^ may potentially serve as a prospective biomarker in elucidating the connection between periodontitis and other systemic diseases. Nonetheless, it is essential to consider that systemic diseases might also impact LMR which should be considered in predicting periodontitis.

## 7 PLATELET TO LYMPHOCYTE RATIO

### 7.1 The basis of PLR

**FIGURE 5:**
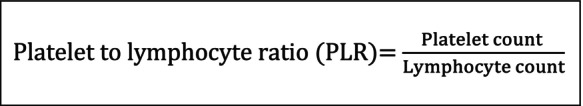
Calculation of the platelet to lymphocyte ratio (PLR).

The PLR is a hematological parameter derived from a standard full blood count. It is calculated by dividing the platelet count by the lymphocyte count (both are measured in cell number/μL, Figure 5). This ratio is emerging as a significant marker in various clinical settings, especially in the context of inflammation, in relation to cancer and cardiovascular diseases.^35^, ^36^, ^37^, ^38^ Furthermore, PLR exhibits greater predictive capability because of its high sensitivity and specificity compared to the sole use of either platelet or lymphocyte counts.^40^ The importance of PLR is based on its ability to reflect the balance between thrombotic risk and immune response. An elevated PLR can indicate a prothrombotic state and/or a suppressed immune response, which is relevant in various pathological conditions. In cardiovascular conditions, PLR is being studied as a potential marker for the severity and prognosis of diseases like CAD.^40^

### 7.2 The use of PLR in cancer

PLR has emerged as a potential prognostic marker in cancer, reflecting the intricate interplay between pro‐tumorigenic and antitumorigenic components of the immune system. The underlying mechanisms involve the role of platelets in promoting tumor‐associated inflammation, angiogenesis, and metastasis, while lymphocytes play a critical role in antitumor immune responses. To date, a large number of reviews and meta‐analyses have been published on the relationship between PLR and different types of cancer. Elevated PLR values are commonly observed across various types of cancer and have been associated with adverse clinical outcomes, including advanced disease stages and reduced overall survival (HR = 1.45, 95% CI = 1.31–1.61), as described in a systematic review and meta‐analysis by Li et al.^196^ Therefore, PLR has been widely accepted as an useful prognostic factor for, that is, lung cancer,^197^ colorectal cancer,^198^ esophageal cancer,^199^ and oral cancer.^200^ However, the utility of PLR as a universal prognostic marker requires more validation across different types of cancer and stages. High PLR levels of >150 are discussed as such a value, but have not been established yet.^196^ In contrast, to the proposed cutoff value for PLR in cancer, there is no cutoff value defined in other diseases and studies indicate that average PLR values vary across different racial backgrounds and different age groups.^90^, ^91^, ^153^, ^173^, ^196^, ^201^, ^202^

### 7.3 Findings from the use of PLR in periodontology

PLR in relationship to periodontal diseases has not been sufficiently addressed in the literature.^173^ Both lymphocyte and platelet levels may be increased as a response to periodontal pathogens.^86^ It can be presumed that after periodontal treatment, the levels respond to it with a decrease^68^, ^74^ PLR is valued for its simplicity, cost‐effectiveness, and accessibility, as it can easily be calculated from routine blood tests. However, its interpretation must be contextualized within the broader clinical picture, as it can be influenced by various factors like infection, systemic inflammation, and hematological disorders.^40^ The combination of PLR and NLR has been identified as effective in reflecting the inflammatory response and is considered as prognostic marker and predictor of systemic diseases. These indices correlate with elevated levels of pro‐inflammatory mediators, attesting to a proinflammatory status.^203^, ^204^, ^205^

Some of the studies published so far have failed to show an association between PLR and periodontitis. The largest GAgP study based on a Chinese population by Lu et al.^91^ which has already been discussed in the paragraph on NLR, did not find differences between patients and controls (periodontitis: mean PLR = 132 vs healthy: mean PLR = 126). The authors suggested that in patients diagnosed with GAgP, the functional activation of platelets may be of greater clinical significance than the numerical count.

These results were confirmed by Mishra et al.^138^ The authors analyzed the PLR which was in contrast to the NLR not associated with severe periodontitis (periodontitis: mean PLR = 144 vs heathy: mean PLR = 134, *p* = 0.574). Furthermore, PLR was not associated with most of the recorded periodontal parameters.

The same study group published a further study with a larger study population with controversial results. While absolute platelet counts (periodontitis: 278 vs heathy: 266) were not significantly higher in the periodontitis group, absolute lymphocyte counts (periodontitis: 1.93 vs heathy: 2.2) were significantly lower. This resulted in a significantly different PLR of 136 in patients with generalized stage III grade C periodontitis compared to 124 in healthy individuals. ROC yielded cutoff values of PLR > 126 in discriminating patients with periodontitis from healthy individuals. Based on the cutoff values, multiple logistic regression analysis reported a lower but significant association of periodontitis with PLR (OR = 2.16, 95% CI = 1.56–3.01) after adjusting for oral hygiene habits, BMI, and total WBC count. Nevertheless, the area under the curve for PLR was only 0.576 and therefore distinctly lower than NLR or SII. Furthermore, the accuracy of both NLR and SII were found to be superior to PLR since PLR exhibits higher sensitivity (64%), specificity (53%), and diagnostic accuracy (59%). As a result, PLR did not improve predictive quality and accuracy of generalized stage III grade C periodontitis diagnosis.^137^

Stronger evidence supporting the hypothesis that periodontitis is associated with PLR was published by Acharya et al.^90^ This study analyzed PLR before and after anti‐infective periodontal therapy. The included study population and the limitations of the study were discussed above in the NLR part. After 1 month, the 30 treated chronic periodontitis patients showed a significant reduction of PLR from 121 to 80. Before treatment, PLR correlated positively with PPD (not CAL; PPD: r = 0.1762, t = 1.1740, *p* = 0.2469). However, after successful treatment, PPD and CAL correlated negatively (PPD: *r* = −0.0612, t = −0.4023, *p* = 0.6894; CAL: r = −0.1578, t = −1.0481, *p* = 0.3005). Furthermore, the included healthy controls had a PLR of 112. That means PLR increases in a periodontal inflammation and a therapeutic intervention had a remarkable influence on PLR.

Another interesting study was published by Torrungruang et al.^141^ including diabetics and periodontitis patients. This cross‐sectional study investigated the relationship between different glycemic status, PLR, and periodontitis in a Thai population. It benefited from its robust sample size of 2036 participants. The study design, the inclusion criteria, and the results between the association of periodontitis and type 2 diabetes are shown above in this review. Between no/mild, moderate, and severe periodontitis patients' smaller differences were observed but not statistically significant. In comparison to subjects with nonsevere periodontitis (encompassing both no/mild and moderate cases), patients with severe periodontitis demonstrated a significantly lower PLR (*p* = 0.015). Furthermore, PLR decreased with significantly worsening glycemic status. When controlling for the severity of periodontitis, PLR was significant negatively associated with diabetes (*p* = 0.007). A possible association of periodontitis with diabetes through the PLR was found to be significant. Analyzing the single components of the PLR revealed a reverse relationship of these components in diabetes or periodontitis. The rather high increase of the lymphocyte counts seems to be of greater importance than the minor decrease in the platelet number. These results were in contrast to the findings by Mishra et al.^137^, ^138^ Potential reasons for this discrepancy could include variations in the study design, the high heterogeneity of the study population (age, gender, and smoking habits), and in blood cell counts in both studies. While the authors mentioned the smoking status, they did not discuss the effects on PLR. The study's cross‐sectional design precludes the elucidation of temporal and longitudinal associations among periodontitis, glycemic status, and in between PLR. The authors concluded, that PLR is an important risk indicator and highly reverse dose‐dependent in patients with/without diabetes and periodontitis.^141^

PLR values presented in these studies are in line with those documented in Korean,^153^ Chinese,^91^ and Nigerian patients.^201^ However, they are notably higher compared to studies from India.^90^, ^202^ This discrepancy may be attributed to regional variations or the inclusion of a younger age group. A recent meta‐analysis that included four^90^, ^91^, ^137^, ^141^ out of five studies^138^ found a mean PLR increase of 1.83 in periodontitis patients compared to control groups, but the result was not statistically significant. One of the studies that included diabetic patients had to be excluded since it had a disproportionate influence on the statistical significance.^141^ After its exclusion, the results showed a much lower heterogeneity. Notably, 25% of the participants in this study exhibited impaired glucose tolerance or type 2 diabetes mellitus, contrasting with other studies investigating PLR that did not include patients with systemic diseases. This exclusion, as suggested by Almășan et al.,^173^ was necessary because it resulted in a more homogeneous health profile which enhances the validity of the meta‐analysis. However, the authors concluded that PLR is not a good single systemic inflammation biomarker for severe periodontitis. More studies are needed to verify the existing results.

## 8 PLATELET DISTRIBUTION WIDTH

### 8.1 The basis of PDW

**FIGURE 6:**
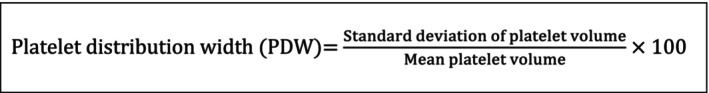
Calculation of the platelet distribution width (PDW).

Larger platelets, characterized by an increased granular content, indicate the potential for a rapid aggregation with collagen. Further, it indicates elevated thromboxane A2 levels and increased expression of glycoprotein Ib and IIb/IIIa receptors compared with smaller platelets.^206^, ^207^ These findings show that mean platelet volume (MPV) and PDW may be influenced by many inflammatory and cardiovascular risk factors.^144^, ^208^ Figure 6 illustrates how DNI is calculated. A study by Zhan et al.^169^ examined gingival biopsies using immunohistochemistry and electron microscopy. They observed a reduction in platelet size in patients with GAgP. Weak negative associations between platelet size and periodontal parameters were detected (*p* ≤ 0.025). Platelet aggregates and adhesion to the endothelium and leukocytes were shown in venules and connective tissues of gingival biopsies. Platelet large cell ratio increased after periodontal therapy (*p* ≤ 0.038). The authors concluded from these results that the reduced platelet size was due to the consumption of large platelets at sites of periodontal inflammation. Additionally, in a study addressing platelet volume indices among patients with CAD and acute myocardial infarction, Khandekar et al.^209^ concluded from the results that MPV and PDW levels are elevated in patients with myocardial infarction and angina pectoris.

### 8.2 The use of PDW in cancer

PDW has been investigated as a potential biomarker in cancer research, reflecting alterations in platelet size and heterogeneity. The specific mechanisms underlying PDW variations in cancer remain complex, involving interactions between platelets and the tumor microenvironment. Studies suggest a nuanced relationship, with both increased and decreased PDW values that were reported in various types of cancer.^210^, ^211^ Elevated PDW levels have been associated with certain types of cancer, that is, breast cancer,^212^ laryngeal cancer,^213^ and colorectal cancer.^214^ A meta‐analysis by Xia et al.^211^ demonstrated strong evidence for high pretreatment PDW levels in association with poor advanced prognosis. High levels of PDW were related to poor overall survival (HR = 1.54, 95% CI = 1.18–2.00), especially for pharyngolaryngeal (HR = 3.06, 95% CI = 1.68–5.57) and breast cancer (HR = 1.21, 95% CI = 1.07–1.36). While PDW shows promise as a diagnostic and prognostic marker, its utility is not universally consistent across all cancer types. Therefore, a precise PDW cutoff value for every cancer or a general cutoff value has not been found yet.

### 8.3 Findings from the use of PDW in periodontology

Two studies evaluated PDW in periodontitis patients. Temelli et al.^139^ included 77 periodontitis patients with and without CAD in a cross‐sectional design. In one out of four groups, a significant difference in PDW could be detected. The level of PDW was significantly higher in the group with both CAD and periodontitis compared with the CAD group without periodontitis. It has to be considered that the small sample size (*n* = 20 in each group) could be a confounder. This association between periodontitis and elevated PDW is further corroborated by statistically significant correlations between PDW and periodontal inflamed surface area (PISA, correlation coefficient = 0.24, *p* = 0.036), respectively, CAL (correlation coefficient = 0.243, *p* = 0.033). Evidence of prothrombotic activation in relation to PDW and periodontitis was also shown in this study. These important findings should be given attention since the authors reported for the first time the connection between CAD and periodontitis through markers such as PISA, MPV, and PDW. The implication that PDW may serve as a valuable prognostic biomarker in patients with cardiovascular disease should be integrated into future research identifying the linkages between these two diseases.

The second study was published by Mutthineni et al.^144^ The authors included an Indian population which is notably limited by a small sample size, encompassing 25 healthy individuals, 25 patients with moderate, and 25 with severe periodontitis. The mean PDW levels showed slight but not significant differences compared to healthy (10.51), moderate (10.61), and severe (10.87) periodontitis. Both studies conclude that PDW can be used as simple, practical, and cost‐effective systemic biomarker for periodontitis. However, there is currently too little evidence for a possible association.

## 9 PLATELETCRIT

### 9.1 The basis of PCT

**FIGURE 7:**
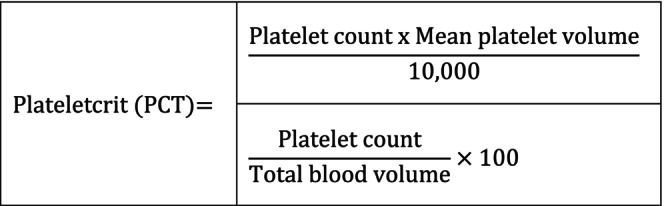
Calculation of the plateletcrit (PCT).

PCT serves as an analytical index that offers insights into the aggregated mass of platelets. There are two ways in which PCT can be calculated, both options are shown in Figure 7.^215^ MPV constitutes a component of PCT but can also be used alone as a biomarker in various inflammatory diseases.^216^ Studies present divergent findings concerning the correlation between MPV and systemic inflammation, documenting either a positive or negative association.^216^, ^217^ Moreover, Ekici et al.^218^ reported a robust association between MPV values and the angiographic severity of CAD. PCT levels fluctuate within the scope of 0.22%–0.24%, making the evaluation of this metric a valuable tool for enhancing the precision of inflammation and the diagnosis of an elevated inflammatory event.^215^ Additionally, PCT has been validated as a dependable marker for diagnostic and therapeutic management of several diseases.^219^, ^220^ Growing evidence confirms that platelet indices, including PCT, disclose significant associations with vascular risk factors.^221^ A retrospective study conducted by Aslan et al.^222^ that incorporated 230 patients with more than 50% stenosis of the carotid artery, observed statistically elevated PCT levels in these patients. Furthermore, the authors suggested that PCT could predict long‐term adverse outcomes and has the potential to be an independent predictive marker for long‐term mortality. Şahin et al.^223^ concluded that PCT serves as an indicator of the numbers of platelets/μl. This deduction was based on their observation that PCT levels were elevated in patients suffering from pulmonary tuberculosis compared to those afflicted with pneumonia. A further investigation indicated a robust association between elevated PCT levels and both saphenous vein disease and slow coronary flow.^224^

### 9.2 The use of PCT in cancer

A paucity of studies has reported on findings regarding PCT across different types of cancer, which may indicate a complex relationship between PCT and tumorigenesis. Studies with elevated PCT levels have been associated with certain malignancies, suggesting potential connections to tumor‐related inflammation, angiogenesis, and platelet activation, that is, in lung cancer,^225^, ^226^ colorectal cancer,^227^, ^228^ and breast cancer.^229^ Hur et al.^226^ described a PCT of >0.2755% as factors for overall survival and disease‐free survival that predicts a poor prognosis for lung cancer, showing hazard ratios of more than 4.18 (95% CI = 1.54–11.34) and 4.07 (95% CI = 1.52–10.94), respectively. Zhu et al.^227^ demonstrated that PCT levels of colorectal cancer patients were associated with tumor size and TNM stages. In addition, another study showed a PCT level of 0.26% in colorectal cancer patients in contrast to 0.22% in healthy controls.^228^ Therefore, elevated PCT levels in individuals who were systemically healthy could indicate an increased susceptibility to systemic diseases. However, conflicting reports also highlight decreased PCT in some cancer studies,^230^, ^231^ indicating potential alterations in platelet production and turnover. The underlying mechanisms maintaining PCT variations in cancer remain intricate and involve interactions between platelets and the tumor microenvironment. While PCT holds promise as a diagnostic and prognostic marker but its utility is not demonstrated yet to be universally usable for all types of cancer.

### 9.3 Findings from the use of PCT in periodontology

Two studies investigated the PCT in periodontitis patients. Ustaoglu et al.^145^ evaluated the PCT in 57 stage III periodontitis patients and 57 healthy controls. Unfortunately, grading, extent, and distribution were not described in this study. Smokers were excluded as smoking was considered a confounder and no significant differences in age and gender between the study groups were detected. MPV was significantly higher in the periodontitis group, platelet counts were higher in the periodontitis group but the difference was not statistically significant. As a result, PCT was found to be significantly elevated in the periodontitis group (periodontitis: mean PCT = 0.223%, healthy controls: mean PCT = 0.196%). Additionally, PCT was positively associated to periodontal clinical parameters such as PPD, CAL, and BOP. These data offer a more precise understanding of platelet mass and activity in periodontal disease. However, the limitation of this study is a small sample size and a cross‐sectional study design.

The second study which investigated the association between PCT and periodontitis was published by Mutthineni et al.,^144^ which was conducted on an Indian population. It is constrained due to its relatively small sample size. Included were 25 healthy, 25 moderate, and 25 severe periodontitis patients. The study did not account for variables such as platelet disorders and the race or gender of the patients, which are factors with additional influence. The mean PCT levels were 0.19% in healthy individuals, 0.3% in those with moderate, and 0.42% in cases with severe periodontitis. As indicated by ANOVA with post hoc Games‐Howell testing, significant differences in PCT levels were observed from healthy to diseased individuals. This was confirmed by one‐sample t‐test showing for healthy and severe periodontitis significant differences. The chronic inflammatory response in periodontitis leads to a tendency for platelet aggregation and to activation, resulting in alterations in platelet size, shape, and number. These changes in platelet indices substantiate their reliability as biomarkers for evaluating both periodontal and various inflammatory diseases.

## 10 SYSTEMIC IMMUNE INFLAMMATION INDEX

### 10.1 The basis of SII

**FIGURE 8:**
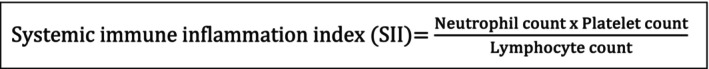
Calculation of the systemic immune inflammation index (SII).

SII is a novel, good, and stable marker, reflecting both immune response and systemic inflammation at low costs, making it a useful indicator for clinical applications.^232^, ^233^, ^234^ This index was developed by Hu et al.^232^ and integrates the analysis of neutrophils, platelets, and lymphocyte counts. Figure 8 illustrates the calculation of the DNI. SII has no universally acknowledged cutoff value, with limited research exploring reference values in healthy adults. Luo et al.^235^ identified a reference value of 334.0 × 10^9^/L (range: 142–804 × 10^9^/L) in a large multicenter study of a Chinese population. Correspondingly, Fei et al.^236^ determined a reference interval for SII in an indigenous Chinese population to be 190.5–760.9 × 10^9^/L. Additionally, a longitudinal population‐based study by Fest et al.^237^ in the Netherlands reported an average SII value of 459 × 10^9^/L. Furthermore, in a recent study investigating the association between SII and psoriasis, it was suggested that a cutoff value of 578.8 × 10^9^/L could indicate psoriasis activation.^238^ Additionally, a large‐scale US study with a 20‐year‐follow‐up including 42 875 adults showed that SII levels of >655 × 10^9^/L had higher all‐cause mortality and cardiovascular mortality than those participants with SII levels <335 × 10^9^/L.^239^

### 10.2 The use of SII in cancer

To date, a large number of reviews and meta‐analyses have been published on the relationship between SII and different types of cancer. Elevated pretreatment SII levels have been observed in various kinds of cancer, reflecting a systemic inflammatory response and an imbalance in the immune milieu. Increased SII values have been associated with poor prognosis in several malignancies, indicating a potential association with tumor aggressiveness and systemic inflammatory conditions. The underlying mechanism involves the role of platelets and neutrophils in promoting tumor‐associated inflammation, angiogenesis, and immune evasion, while decreased lymphocyte counts contribute to compromised antitumor immune responses. Therefore, SII has been widely accepted as a useful prognostic factor which has been presented in meta‐analysis for head and neck cancer (HR for poorer overall survival = 2.09, 95% CI = 1.62–2.70; cutoff value ≥520 × 10^9^/L),^240^ breast cancers (HR for poorer overall survival = 2.12, 95% CI = 1.61–2.79; cutoff value >600 × 10^9^/L),^241^ lung cancer (HR for poorer overall survival = 1.52, 95% CI = 1.15–2.00; cutoff value ≥700 × 10^9^/L),^242^ pancreatic cancer (HR for poorer overall survival = 1.55, 95% CI = 1.34–1.78; cutoff value ≥900 × 10^9^/L),^243^ and gastric cancer (HR for poorer overall survival = 1.40, 95% CI = 1.08–1.81; cutoff value ≥600 × 10^9^/L).^244^ A recent study by Nøst et al.^245^ used a prospective UK biobank cohort of 442 115 participants to investigate a possible association between SII, NLR, PLR, and LMR aiming to assess the risks of 17 cancer types by estimation of the specific hazard ratios. The authors observed a positive risk association for seven out of 17 cancers with SII, NLR, PLR, and a negative association with LMR. SII exhibited the strongest associations with lung and colorectal cancer risk. In conclusion, the elevated risk observed in the year preceding diagnosis may signify a systemic immune reaction to an existing but asymptomatic cancer. SII could serve as a biomarker for the risk of cancer incidence, offering the potential for early disease detection in the final year preceding clinical diagnosis. However, the utility of SII as a universal prognostic marker requires further validation across different types of cancer and stages because cutoff values varied widely in studies and meta‐analyses.

### 10.3 Findings from the use of SII in periodontology

In patients with rheumatoid arthritis, Liu et al.^246^ observed a nonlinear association to SII. Based on this finding, two recent studies investigated the hypothesis that a similar nonlinear relationship between SII and periodontitis may exist,^137^, ^149^ because lower neutrophil recruitment in periodontal tissue is associated with significant bone loss as demonstrated by Hajishengallis.^247^

Periodontitis can be regarded as a model of low‐grade systemic inflammation that exhibits similar pathophysiology as in rheumatoid arthritis. SII also is used for various systemic diseases such as diabetes mellitus,^248^ CAD,^44^, ^249^ hypertension,^250^ rheumatoid arthritis,^246^, ^251^ psoriasis,^252^ and psoriatic arthritis.^253^ Thus, SII may serve as a potential indicator of systemic inflammatory response in patients in both periodontitis as well as the systemic diseases listed above.

**FIGURE 9:**
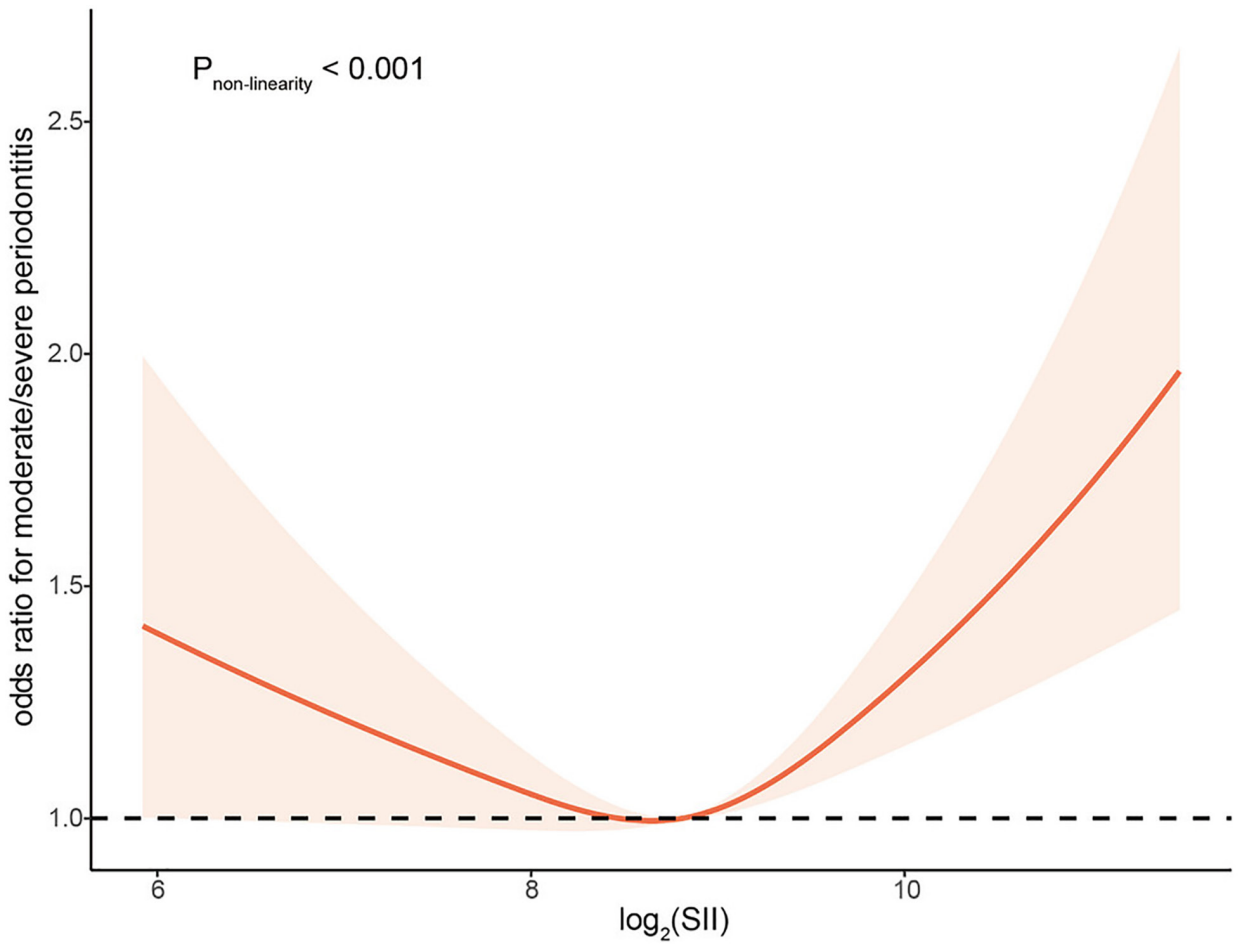
The relationship between systemic immune inflammation index (SII) and moderate/severe periodontitis follows a J‐shaped curve. Figure was published by Cao et al.^149^ and reprinted here with editorial permission.

A cross‐sectional study by Cao et al.^149^ included a large population of 10 301 US adults based on three cycles of survey data obtained from the National Health and Nutrition Examination Survey (NHANES) 2009–2014. The NHANES is a comprehensive multistage, stratified, clustered probability sampling research initiative to assess the health and nutritional landscape of the US civilian population.^254^ The individuals underwent a full‐mouth periodontal examination and the SII was categorized into five levels: log_2_(SII): ≤8.21, >8.21–8.81, >8.81–9.42, >9.42–9.75, and >9.75. Statistical comparisons among these levels revealed significant variations in numerous parameters encompassing average age, gender, ethnicity, marital status, smoking habit, obesity rates, diabetes prevalence, hypertension rates, mean CAL, mean PPD, number of sites PPD ≥4 mm, number of sites CAL ≥3 or 5 mm, and the prevalence of periodontitis. The association between SII and periodontitis demonstrates a J‐shaped curve (*p* for nonlinearity <0.001), with an inflection point value of log_2_(SII) at 8.66 (Figure 9). When log_2_(SII) is ≤8.66, each unit increment in log_2_(SII) reduces the risk of moderate to severe periodontitis by 17% (OR = 0.83, 95% CI = 0.69–0.999). However, post this threshold (log_2_(SII) >8.66), the risk amplifies by 19% for each unit increase (OR = 1.19, 95% CI = 1.02–1.38). Interestingly, these individuals with moderate to severe periodontitis with an SII beyond the inflection point had lower SII values compared to no/mild periodontitis patients at log_2_(SII) ≤8.66. Yet, for those with log_2_(SII) >8.66, the SII values increased, indicating heightened host immune activity. An explanation for this could be that the immune responses strongly vary between the different occurrences of periodontitis ranging from moderate to severe.^255^

A preceding investigation established a similar but inverse U‐shaped curve between SII and the prognosis of gastric cancer.^256^ Therefore, integrating anti‐inflammatory therapies alongside conventional periodontal therapy^257^, ^258^ might be particularly advantageous for patients with log_2_(SII) values of >8.66. On the other hand, stimulating the immune mechanism during periodontal therapy could prove to be beneficial for those with log_2_(SII) values of ≤8.66. Additionally, non‐Hispanic Whites showed a J‐shaped SII and periodontitis association, while both Mexican Americans and non‐Hispanic Blacks showcased a linear association. This divergence might arise from inherent ethnical differences in inflammatory response and genetic predispositions.^259^

SII was significantly higher in periodontitis patients (724 × 10^9^/L) compared to the periodontally healthy group (538 × 10^9^/L). ROC yielded cutoff values of > 591 and a sensitivity of 81%, a specificity of 77%, and a diagnostic accuracy of 79%. Thus, SII was highly associated with severe periodontitis in young adults after adjustments (OR = 11.86, 95% CI = 7.99–17.59). The SII exhibited reasonable predictive accuracy for the identification of severe periodontitis in young adults and may offer insights for disease diagnosis and therapeutic interventions. Through pairwise comparison, it was observed that the SII was not statistically distinct from the NLR but both were superior when compared to PLR.

The observed discrepancies of SII values between the studies including periodontitis and other inflammatory diseases may also be attributable to the type of automated blood analyzer and the differences in ethnicity, a factor that requires consideration in future research. As a result, SII could be used as an adjunctive diagnostic tool in conjunction with standard clinical assessments for the identification and monitoring of patients with severe periodontitis. However, more studies are needed to verify the present results.

## 11 RED BLOOD CELL DISTRIBUTION WIDTH

### 11.1 The basis of RDW

**FIGURE 10:**
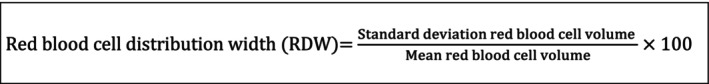
Calculation of the red blood cell distribution width (RDW).

In this review, RDW was selected because it is a robust, independent diagnostic, and prognostic indicator for various diseases. RDW quantifies the variation in the size of erythrocytes. It is usually expressed as a percentage, is calculated as part of a standard full blood test, and is relatively inexpensive. It is a coefficient of variation of red blood cell volume and its calculation is shown in Figure 10.^260^ MCV is the average volume of individual red blood cells. The standard deviation in this context measures how much the sizes of the erythrocytes vary around this mean volume. A higher RDW value indicates a greater variation in size among the erythrocytes in the sample, which is termed anisocytosis. This can be indicative of various medical conditions such as early detection of iron deficiency and differentiating iron‐deficiency states, in which the RDW level is increased. Nevertheless, RDW may be normal in anemia of chronic disease.^261^, ^262^ However, several investigators have observed that while RDW may be elevated in patients with iron‐deficiency anemia compared to those with anemia of chronic disease, it demonstrates limited efficacy in distinguishing between these two conditions.^263^, ^264^

Normal RDW values typically range between 11% and 15%.^260^, ^265^ RDW is often used in conjunction with other hematological measurements, like the previously discussed parameters, to provide a more comprehensive understanding of hematological disorders and the overall health of the erythrocyte's population. Studies demonstrated that RDW is a predictor of adverse outcomes in patients with heart failure, CAD, stroke, hypertension, and as a novel marker for erythropoiesis.^266^, ^267^, ^268^, ^269^ Therefore, RDW is an accepted prognostic index for cardiovascular diseases.^269^, ^270^

### 11.2 The use of RDW in cancer

RDW has been investigated as a potential biomarker for different kinds of cancer, indicating potential diagnostic and prognostic significance. The link between increased RDW and cancer may be attributed to the inflammatory and oxidative stress responses commonly associated with malignancies, influencing erythropoiesis and red blood cell morphology. Additionally, cancer‐related anemia, characterized by altered red blood cell dynamics, can contribute to elevated RDW values. Several systematic reviews and meta‐analyses reported an independent association between elevated RDW and poorer outcomes in cancer patients, emphasizing its potential as a prognostic indicator. Meta‐analyses showed an association between high pretreatment RDW levels and worse overall survival in breast cancer (HR = 2.12, 95% CI = 1.47–3.08),^271^ glioma (HR = 1.40, 95% CI = 1.13–1.74),^272^ lung cancer (HR = 1.55, 95% CI = 1.26–1.92),^273^ esophageal cancer (RDW cutoff value >13%, HR = 1.45, 95% CI = 1.13–1.76),^274^ gastric cancer (RDW cutoff value >14%, HR = 1.79, 95% CI = 1.21–2.66),^275^ colorectal cancer (HR = 1.99, 95% CI = 1.59–2.49),^276^ and urological cancer (HR = 1.52, 95% CI = 1.27–1.82).^277^ The review by Wang et al.^278^ provides a good overview. The authors summarize that high pretreatment RDW levels with cutoff values >14% or > 15% are associated with poor survival outcomes in various malignancies. The use of RDW as a stand‐alone marker for cancer diagnosis or prognosis requires further validation because most of different types of cancer lead to controversial results.

### 11.3 Findings from the use of RDW in periodontology

RDW is one of the most frequently examined systemic indices in periodontitis patients. Bhattacharya et al.^131^ and Sridharan et al.^148^ demonstrated a significant higher RDW level in periodontitis patients compared to the healthy controls. Furthermore, Sridharan et al.^148^ evaluated RDW in 40 hypertensive and 40 nonhypertensive patients with and without periodontitis which were also described above. This study demonstrated that both hypertension and periodontitis independently influence RDW. Furthermore, the study showed that age was significantly related to RDW, but not gender. The combination of periodontitis and systemic hypertension leads to a substantial increase in RDW. Regarding mean CAL, PPD, plaque index, gingival index (as dependent variables), and RDW, statistically significant association for all were demonstrated. These findings also suggest a potential cumulative impact of periodontitis on RDW in hypertensive patients with periodontitis. As a result, it is imperative to underscore rigorous treatment of periodontitis in patients with hypertension, as this could be pivotal in averting detrimental cardiovascular consequences.^279^, ^280^ This was confirmed by Bhattacharya et al.^131^ in an Indian population, where RDW was found to be significantly higher in periodontitis patients.

The remaining four studies did not find an association. Anand et al.^146^ investigated the relationship between 64 GAgP patients and 58 healthy controls by analysis of the erythrocyte count, hemoglobin levels, and the RDW. As a confounder, there was a significant difference between the two groups concerning smoking habits. However, after adjusting for variables like age, gender, smoking, and BMI through logistic regression analysis, a significant association was identified between GAgP and decreased erythrocyte counts but not for RDW.

Previous findings suggested an improvement of erythrocyte count and hemoglobin post periodontal treatment in anemic patients. This leads to the hypothesis that untreated periodontitis might play a role in reducing total erythrocyte counts.^281^ Studies have shown that patients with GAgP often exhibit a systemic inflammatory response by elevated serum levels of proinflammatory cytokines.^282^, ^283^ This systemic immune response in GAgP could probably explain the observed decrease in erythrocyte counts and hemoglobin levels. Conversely, while the mean RDW value for patients with GAgP was marginally higher than that of the control group (15.27% compared to 15.16%), this difference lacked statistical significance. It is important to note that the demographic features of the sampled group, encompassing socioeconomic status, tobacco and alcohol consumption patterns, dietary and oral care habits, might diverge from general population.

A study by Ustaoglu et al.^145^ evaluated RDW in 57 stage III periodontitis patients and 57 healthy controls. The study design is described above in this review. There were no statistically significant differences between periodontitis patients and healthy controls with a RDW value of 14.6 and 15.2, respectively. Consequently, no association was found between RDW and periodontal clinical parameters. These findings confirm the results of a previous study by Anand et al.^146^

One study evaluated RDW in periodontitis and nonperiodontitis patients with and without CAD. Temelli et al.^139^ included 77 patients in a cross‐sectional design. The fact that nonperiodontitis patients are gingivitis patients with a reduced periodontium should be considered when interpreting the results. Significant elevated levels of RDW were observed in the group with periodontitis (without CAD) relative to the group without both periodontitis and CAD, but the sample size was small (*n* = 21 vs *n* = 16). Additionally, a significant positive association was observed between PPD and RDW in the two groups with and without periodontitis and without CAD. This observed associations may suggest that the presence of periodontitis and its inflammatory response could lead to modifications in the size of peripheral blood cells, thereby conferring a heightened susceptibility to cardiovascular diseases, as discussed above in this review addressing platelets. Therefore, a study by López et al.^147^ is of importance. 16 years old periodontitis patients with CAL ≥3 mm (mean 4.1% of sites per person) and 16 years old healthy controls were included (*n* = 87 vs *n* = 73) based on a Chilean population. The RDW was not significantly deviated between periodontitis patients (14.40%) and healthy controls (14.38%). Even a sub‐analysis of the periodontal parameters could not find any correlation. This implies that the inflammatory load of periodontitis in young patients fails to significantly impact RDW. As a conclusion, the age of patients should be more considered in studies. This conclusion is supported by a recent meta‐analysis including four^139^, ^145^, ^146^, ^148^ out of six studies presented in the actual review. Mean RDW higher by 0.1 in the periodontitis group compared to the control group was found, but lacking statistical significance.^173^

In this review, the majority of studies did not yield statistically significant evidence to confirm an association between RDW and periodontitis, as the findings were heterogeneous and characterized by conflicting results regarding the direction of this association.

## 12 LIMITATIONS OF STUDIES RELATED TO SYSTEMIC INFLAMMATION INDICES IN PERIODONTITIS

There are several limitations of the reviewed studies such as study design, region, sample size, classification of disease and health, age, gender, plaque score, smoking status, systemic disease as well as time between blood collection and analysis. The designs of nearly all studies showed weaknesses that led to a low level of evidence in the results for these indices in periodontitis. The observational design of the studies just permits to identify associations.

Only one study, conducted by Acharya et al.,^90^ evaluated the impact of periodontal treatment on NLR as well as PLR and observed a significant reduction in their posttreatment values. Nearly all studies were performed in Asia, only one study was carried out in the USA and another one was from Chile. No study has yet been conducted in Europe. This suggests that the outcomes could potentially exhibit variations attributable to racial differences, as well as regional disparities within the same ethnical group. Another limitation was the small sample size, more than half of the discussed studies included less than 150 participants.

The classification of periodontitis differed between studies;^52^, ^130^, ^132^, ^136^ in addition, authors used different criteria to identify the disease as opposed to official classifications. Furthermore, studies worked with different severity stages of periodontitis. The selection of periodontitis and healthy participants was inconsistent as well as the criteria for periodontal health. Also, exclusion criteria of systemic diseases differed between studies. Participants were often recruited from a single outpatient department, for example, department of periodontology, internal medicine, or cardiology.

A limited number of studies did not elaborate on the potential effects of specific sociodemographic factors such as education, occupation, and socioeconomic status on blood cell ratios and periodontal conditions. Adult individuals more likely exhibit multimorbidity. In 50% of periodontitis patients, comorbidity occurs.^284^, ^285^ In these concurrent inflammatory conditions and additional influential factors are more likely present in adult compared to adolescents. Consequently, comparisons between young patients with periodontitis and healthy controls are likely less impacted by confounding factors. It is reasonable to anticipate that differences in blood parameters and inflammation indices between individuals with and without periodontitis would be more obvious among younger subjects. Only one study by López et al.,^147^ compared general hematological values in adolescents based on their periodontal status.

A study on a South Korean population^153^ and one in a Chinese Han population^286^ demonstrated a positive association between NLR and factors such as age and gender in systemically healthy adults. In contrast, a study in an Indian population observed no significant differences in blood leukocyte ratios relative to age.^202^ Therefore, the potential influence of age must be taken into account when considering blood cell ratios as predictors for the manifestation and progression of severe periodontitis. Just a limited number of studies matched the groups for age and gender.

This review should also acknowledge that individuals with periodontitis often have poor dental care, leading to an increased plaque accumulation, which in turn may amplify the extent of bacteremia which have been reported to occur transiently, that is, after oral examination and most likely several times a day.^13^, ^14^ The recruitment of leukocytes, especially polymorphonuclear neutrophils, intensifies the further recruitment of neutrophils in a positive feedback loop during episodes of bacteremia in periodontitis or when lipopolysaccharides infiltrate the systemic circulation.^13^ Consequently, poor oral hygiene practices may likely influence different inflammation indices. Only a few studies considered this aspect and assessed the plaque score as well.^133^, ^137^

A recent systematic review and meta‐analysis investigated the impact of smoking on periodontitis, revealing that smoking exacerbates periodontitis.^287^ Utilizing an innovative artificial intelligence‐based network analysis, the authors identified systemic multimorbidity clusters in individuals with periodontitis and examined factors that may affect the severity of these clusters. They reported that arthritis, hypertension, and obesity had the most significant influence on multimorbidity clusters in subjects with periodontitis. Furthermore, diabetes was found to be more prevalent among those who had increased CAL. A subsequent study indicated that in adults with severe periodontitis, the smoking status affected the clustering patterns of diabetes and cancer.^284^ This is confirmed by a recent study that classifies demographic data, lifestyle factors, systemic diseases, and periodontitis into different clusters and demonstrated an overlap of the clusters. Therefore, it can be assumed that there is an overlap in pathophysiology which leads to the suggestion, that periodontitis can be regarded as a part of multimorbidity, manifesting as a systemic disease that concurrently affects certain patients.^285^ In conclusion, periodontitis, systemic diseases, and the other factors listed above may affect inflammation indices.^284^, ^285^

Furthermore, the duration between blood sample collection and analysis is deemed critical, as the composition of blood cells may undergo alterations or cells may be destroyed upon the time lapse between these procedures.^153^, ^288^, ^289^ Only few studies reported exact times between sampling and analysis of maximum of 30 min. This time frame is recommended by several previous studies.^290^ Finally, a blood cell analyzer from the Company Sysmex Corporation (Kobe, Japan) was often used for blood cell analysis. However, different analyzers and measurement techniques influences the outcomes of the results of the blood cell parameters.

In adult study populations, high variations in blood parameters are to be expected. In some studies, even as elevated described, values did not exceed normal reference values even considering that the range of normal values is based of more than 10 000 people which possibly exhibit morbidities.^13^ However, a challenge in analyzing hematological values in adults with periodontitis is their potential to be influenced by a range of factors beyond periodontal disease. Adolescents with periodontitis tend to represent a more homogeneous group, less impacted by other latent infectious or inflammatory factors. Therefore, investigations including adults exhibit a higher complexity. If the hemogram of adolescents with periodontitis deviates from the norm reference, it could more convincingly be attributed to periodontal status rather than being a coincidental finding. Nevertheless, we need to address the (older) adult population with inflammatory risks.

## 13 CONCLUSIONS AND OUTLOOK

Periodontitis is mainly quantified based on the severity and extent of attachment loss and/or PPD, in addition with BOP. This includes signs of the manifestations of past disease like attachment and bone loss, offering minimum insight into current activity of the disease. To assess present and future disease activity, local and systemic inflammation indices are predestined as tools for precise detection and quantification and for monitoring the risk of future development of periodontitis. Furthermore, inflammation indices are possible parameters to evaluate the impact of periodontitis on systemic health and may provide a tool to quantify the influence of periodontitis on systemic diseases and vice versa.

This is the first review that summarizes current scientific evidence and provides an insight on the possible link between periodontal disease and systemic inflammation indices that are used in cancer diagnosis, monitoring, and management. The great advantage of the discussed systemic indices is that they are based on frequently used blood analysis in daily clinical practice that are used worldwide. They are easy to obtain, fast, cheap, and reliable and thus accessible even for patients residing in low‐ and middle‐income countries. A draw of a tube of blood is standard and routine globally.

Evidence exists for an association between the systemic indices NLR, PLR, PCT, LMR, DNI, and SII and periodontitis, but for RDW and PDW, there was only weak evidence. However, there were weaknesses in methodological quality of the presented and discussed studies. One single blood index in predicting severe periodontitis hardly seems sufficient. On the other hand, the evidences described in this review suggest that periodontitis should be defined as inflammatory systemic disease since it clearly affects the blood inflammation indices NLR, PLR, PCT, LMR, DNI, and SII similarly to other systemic diseases.

The following recommendations should be considered for future studies^291^:

First, studies need to be performed including larger populations and in different continents like Europe or Africa in order to evaluate their global validity. The 2017 classification of periodontitis for these studies should be used more consistently.^1^, ^52^ Furthermore, confounders such as age (as young as possible for low inflammaging), gender, smoking status, and racial matched cohorts should be taken account for. It would be useful if studies including more periodontal inflammation indices such as PISA, dentogingival epithelial surface area, periodontal epithelial surface area, and also more current systemic indices such as Extended Inflammation Parameter.^292^, ^293^, ^294^

In addition, the evaluation of the combination of different indices may reflect the balance between host immune and inflammatory conditions more precisely since more components of the immune response would be integrated.

Second, standardization and validation of sampling methods, storage conditions, and analytical techniques, along with examiner calibration are crucial to provide more reliability and clinical applicability of the methods.

Third, the establishment of a causal relationship between inflammation indices and periodontitis is not feasible using only a cross‐sectional study design. Consequently, longitudinal multicenter studies are required. These studies should monitor the long‐term status of chronic inflammation at different time points pre‐ and posttreatment. In addition, these studies may determine whether these findings actually illustrate the relationship between periodontitis and both local and systemic inflammation indices, based on the previously mentioned inclusion criteria.

Fourth, a comprehensive investigation of the underlying biological mechanisms of the link between periodontitis and elevated inflammation indices is essential. In patients with concurrent periodontitis and comorbidities, inflammation indices may reflect the combined effects of periodontitis and systemic diseases. Therefore, it is crucial to consider potential confounders such as systemic conditions and diseases that may influence these values. However, these observations could indicate that inflammation indices might serve as link between periodontal and systemic inflammatory diseases. In particular, to date, no study has been conducted in periodontitis patients with cancer in which the inflammation indices and their changes during therapy were examined.

Interventional studies are required to substantiate possible associations. This will help to elucidate a possible causal bidirectional relation between periodontitis and local as well as systemic inflammation indices. The understanding of the biological mechanisms and principles that determine this complex interplay could support substantial advancements in personalized diagnostics, prognostics, and therapeutic management. Inflammation indices may serve as prognostic markers for predicting future destructive events and assessing the efficacy of treatments at a systemic level. Additionally, standardized use of inflammatory markers may facilitate uniform periodontal diagnosis with severity staging and grading. The development and application of biological tools for diagnostics and the assessment of treatment outcomes represents a key step to the growing medicalization of periodontology.

## CONFLICT OF INTEREST STATEMENT

The authors have stated explicitly that there are no conflicts of interest in connection with this article.

## Data Availability

Data sharing not applicable to this article as no data sets were generated or analyzed during this study.

## References

[1] Tonetti MS , Greenwell H , Kornman KS . Staging and grading of periodontitis: framework and proposal of a new classification and case definition. J Clin Periodontol. 2018;45(Suppl 20):S149‐S161. 29926495 10.1111/jcpe.12945

[2] Bartold PM , Van Dyke TE . Periodontitis: a host‐mediated disruption of microbial homeostasis. Unlearning learned concepts. Periodontol 2000. 2013;62(1):203‐217. 23574467 10.1111/j.1600-0757.2012.00450.x PMC3692012

[3] Hajishengallis G , Darveau RP , Curtis MA . The keystone‐pathogen hypothesis. Nat Rev Microbiol. 2012;10(10):717‐725. 22941505 10.1038/nrmicro2873 PMC3498498

[4] Meyle J , Chapple I . Molecular aspects of the pathogenesis of periodontitis. Periodontol 2000. 2015;69(1):7‐17. 26252398 10.1111/prd.12104

[5] Van Dyke TE , Bartold PM , Reynolds EC . The nexus between periodontal inflammation and Dysbiosis. Front Immunol. 2020;11:511. 32296429 10.3389/fimmu.2020.00511 PMC7136396

[6] Bartold PM , Van Dyke TE . Host modulation: controlling the inflammation to control the infection. Periodontol 2000. 2017;75(1):317‐329. 28758299 10.1111/prd.12169

[7] Cekici A , Kantarci A , Hasturk H , Van Dyke TE . Inflammatory and immune pathways in the pathogenesis of periodontal disease. Periodontol 2000. 2014;64(1):57‐80. 24320956 10.1111/prd.12002 PMC4500791

[8] Walther K‐A , Gonzales JR , Gröger S , et al. The role of polymorphisms at the Interleukin‐1, Interleukin‐4, GATA‐3 and Cyclooxygenase‐2 genes in non‐surgical periodontal therapy. Int J Mol Sci. 2022;23(13):7266. 35806269 10.3390/ijms23137266 PMC9266438

[9] Schaefer AS . Genetics of periodontitis: discovery, biology, and clinical impact. Periodontol 2000. 2018;78(1):162‐173. 30198130 10.1111/prd.12232

[10] Loos BG , Van Dyke TE . The role of inflammation and genetics in periodontal disease. Periodontol 2000. 2020;83(1):26‐39. 32385877 10.1111/prd.12297 PMC7319430

[11] Rahim‐Wöstefeld S , El Sayed N , Weber D , et al. Tooth‐related factors for tooth loss 20 years after active periodontal therapy – a partially prospective study. J Clin Periodontol. 2020;47(10):1227‐1236. 32696485 10.1111/jcpe.13348

[12] Rosales C . Neutrophil: a cell with many roles in inflammation or several cell types? Front Physiol. 2018;9:113. 29515456 10.3389/fphys.2018.00113 PMC5826082

[13] Loos BG . Systemic markers of inflammation in periodontitis. J Periodontol. 2005;76(11 Suppl):2106‐2115. 10.1902/jop.2005.76.11-S.2106 16277583

[14] Han YW , Wang X . Mobile microbiome: oral bacteria in extra‐oral infections and inflammation. J Dent Res. 2013;92(6):485‐491. 23625375 10.1177/0022034513487559 PMC3654760

[15] Hujoel PP , White BA , García RI , Listgarten MA . The dentogingival epithelial surface area revisited. J Periodontal Res. 2001;36(1):48‐55. 11246704 10.1034/j.1600-0765.2001.00011.x

[16] Machado V , Botelho J , Escalda C , et al. Serum C‐reactive protein and periodontitis: a systematic review and meta‐analysis. Front Immunol. 2021;12:706432. 34394107 10.3389/fimmu.2021.706432 PMC8355591

[17] Luthra S , Orlandi M , Hussain SB , et al. Treatment of periodontitis and C‐reactive protein: a systematic review and meta‐analysis of randomized clinical trials. J Clin Periodontol. 2023;50(1):45‐60. 35946825 10.1111/jcpe.13709 PMC10087558

[18] de Queiroz AC , Taba M Jr , O'Connell PA , et al. Inflammation markers in healthy and periodontitis patients: a preliminary data screening. Braz Dent J. 2008;19(1):3‐8. 19031648 10.1590/s0103-64402008000100001

[19] Papapanou PN , Susin C . Periodontitis epidemiology: is periodontitis under‐recognized, over‐diagnosed, or both? Periodontol 2000. 2017;75(1):45‐51. 28758302 10.1111/prd.12200

[20] Pessoa L , Aleti G , Choudhury S , et al. Host‐microbial interactions in systemic lupus erythematosus and periodontitis. Front Immunol. 2019;10:2602. 31781106 10.3389/fimmu.2019.02602 PMC6861327

[21] Cecoro G , Annunziata M , Iuorio MT , Nastri L , Guida L . Periodontitis, low‐grade inflammation and systemic health: a scoping review. Medicina (Kaunas). 2020;56(6):272. 32486269 10.3390/medicina56060272 PMC7353850

[22] Nibali L , D'Aiuto F , Griffiths G , Patel K , Suvan J , Tonetti MS . Severe periodontitis is associated with systemic inflammation and a dysmetabolic status: a case‐control study. J Clin Periodontol. 2007;34(11):931‐937. 17877746 10.1111/j.1600-051X.2007.01133.x

[23] Moutsopoulos NM , Madianos PN . Low‐grade inflammation in chronic infectious diseases: paradigm of periodontal infections. Ann N Y Acad Sci. 2006;1088:251‐264. 17192571 10.1196/annals.1366.032

[24] Pink C , Kocher T , Meisel P , et al. Longitudinal effects of systemic inflammation markers on periodontitis. J Clin Periodontol. 2015;42(11):988‐997. 26472626 10.1111/jcpe.12473

[25] Larmann J , Handke J , Scholz AS , et al. Preoperative neutrophil to lymphocyte ratio and platelet to lymphocyte ratio are associated with major adverse cardiovascular and cerebrovascular events in coronary heart disease patients undergoing non‐cardiac surgery. BMC Cardiovasc Disord. 2020;20(1):230. 32423376 10.1186/s12872-020-01500-6 PMC7236311

[26] Zhang S , Diao J , Qi C , et al. Predictive value of neutrophil to lymphocyte ratio in patients with acute ST segment elevation myocardial infarction after percutaneous coronary intervention: a meta‐analysis. BMC Cardiovasc Disord. 2018;18(1):75. 29716535 10.1186/s12872-018-0812-6 PMC5930503

[27] Haybar H , Pezeshki SMS , Saki N . Evaluation of complete blood count parameters in cardiovascular diseases: an early indicator of prognosis? Exp Mol Pathol. 2019;110:104267. 31194963 10.1016/j.yexmp.2019.104267

[28] Angkananard T , Anothaisintawee T , McEvoy M , Attia J , Thakkinstian A . Neutrophil lymphocyte ratio and cardiovascular disease risk: a systematic review and meta‐analysis. Biomed Res Int. 2018;2018:2703518. 30534554 10.1155/2018/2703518 PMC6252240

[29] Duman TT , Aktas G , Atak BM , Kocak MZ , Erkus E , Savli H . Neutrophil to lymphocyte ratio as an indicative of diabetic control level in type 2 diabetes mellitus. Afr Health Sci. 2019;19(1):1602‐1606. 31148989 10.4314/ahs.v19i1.35 PMC6531946

[30] Hussain M , Babar MZM , Akhtar L , Hussain MS . Neutrophil lymphocyte ratio (NLR): a well assessment tool of glycemic control in type 2 diabetic patients. Pak J Med Sci. 2017;33(6):1366‐1370. 29492060 10.12669/pjms.336.12900 PMC5768826

[31] Mertoglu C , Gunay M . Neutrophil‐lymphocyte ratio and platelet‐lymphocyte ratio as useful predictive markers of prediabetes and diabetes mellitus. Diabetes Metab Syndr. 2017;11(Suppl 1):S127‐s131. 28017281 10.1016/j.dsx.2016.12.021

[32] Wang SY , Shen TT , Xi BL , Shen Z , Zhang X . Vitamin D affects the neutrophil‐to‐lymphocyte ratio in patients with type 2 diabetes mellitus. J Diabetes Investig. 2021;12(2):254‐265. 10.1111/jdi.13338 PMC7858138 32593190

[33] Dolan RD , Lim J , McSorley ST , Horgan PG , McMillan DC . The role of the systemic inflammatory response in predicting outcomes in patients with operable cancer: systematic review and meta‐analysis. Sci Rep. 2017;7(1):16717. 29196718 10.1038/s41598-017-16955-5 PMC5711862

[34] Winther‐Larsen A , Aggerholm‐Pedersen N , Sandfeld‐Paulsen B . Inflammation scores as prognostic biomarkers in small cell lung cancer: a systematic review and meta‐analysis. Syst Rev. 2021;10(1):40. 33509254 10.1186/s13643-021-01585-w PMC7844954

[35] Thomas MR , Storey RF . The role of platelets in inflammation. Thromb Haemost. 2015;114(3):449‐458. 26293514 10.1160/TH14-12-1067

[36] Sreeramkumar V , Adrover JM , Ballesteros I , et al. Neutrophils scan for activated platelets to initiate inflammation. Science. 2014;346(6214):1234‐1238. 25477463 10.1126/science.1256478 PMC4280847

[37] Wang X , Meng H , Xu L , Chen Z , Shi D , Lv D . Mean platelet volume as an inflammatory marker in patients with severe periodontitis. Platelets. 2015;26(1):67‐71. 24499137 10.3109/09537104.2013.875137

[38] Budzianowski J , Pieszko K , Burchardt P , Rzeźniczak J , Hiczkiewicz J . The role of hematological indices in patients with acute coronary syndrome. Dis Markers. 2017;2017:3041565. 29109595 10.1155/2017/3041565 PMC5646322

[39] Templeton AJ , Ace O , McNamara MG , et al. Prognostic role of platelet to lymphocyte ratio in solid tumors: a systematic review and meta‐analysis. Cancer Epidemiol Biomarkers Prev. 2014;23(7):1204‐1212. 24793958 10.1158/1055-9965.EPI-14-0146

[40] Kurtul A , Ornek E . Platelet to lymphocyte ratio in cardiovascular diseases: a systematic review. Angiology. 2019;70(9):802‐818. 31030530 10.1177/0003319719845186

[41] Mano Y , Yoshizumi T , Yugawa K , et al. Lymphocyte‐to‐monocyte ratio is a predictor of survival after liver transplantation for hepatocellular carcinoma. Liver Transpl. 2018;24(11):1603‐1611. 29893464 10.1002/lt.25204

[42] Watanabe K , Yasumoto A , Amano Y , et al. Mean platelet volume and lymphocyte‐to‐monocyte ratio are associated with shorter progression‐free survival in EGFR‐mutant lung adenocarcinoma treated by EGFR tyrosine kinase inhibitor. PLoS One. 2018;13(9):e0203625. 30192878 10.1371/journal.pone.0203625 PMC6128600

[43] Shimura T , Shibata M , Gonda K , et al. Prognostic impact of preoperative lymphocyte‐to‐monocyte ratio in patients with colorectal cancer with special reference to myeloid‐derived suppressor cells. Fukushima J Med Sci. 2018;64(2):64‐72. 30012939 10.5387/fms.2018-10 PMC6141447

[44] Sanz M , Del Castillo AM , Jepsen S , et al. Periodontitis and cardiovascular diseases. Consensus report. Glob Heart. 2020;15(1):1. 32489774 10.5334/gh.400 PMC7218770

[45] Gopinath D , Kunnath Menon R , Veettil K , George Botelho M , Johnson NW . Periodontal diseases as putative risk factors for head and neck cancer: systematic review and meta‐analysis. Cancers (Basel). 2020;12(7):1893. 32674369 10.3390/cancers12071893 PMC7409086

[46] Hibino S , Kawazoe T , Kasahara H , et al. Inflammation‐induced tumorigenesis and metastasis. Int J Mol Sci. 2021;22(11):5421. 34063828 10.3390/ijms22115421 PMC8196678

[47] Stefaniuk P , Szymczyk A , Podhorecka M . The neutrophil to lymphocyte and lymphocyte to monocyte ratios as new prognostic factors in hematological malignancies – a narrative review. Cancer Manag Res. 2020;12:2961‐2977. 32425606 10.2147/CMAR.S245928 PMC7196794

[48] Templeton AJ , McNamara MG , Šeruga B , et al. Prognostic role of neutrophil‐to‐lymphocyte ratio in solid tumors: a systematic review and meta‐analysis. J Natl Cancer Inst. 2014;106(6):dju124. 24875653 10.1093/jnci/dju124

[49] Buonacera A , Stancanelli B , Colaci M , Malatino L . Neutrophil to lymphocyte ratio: an emerging marker of the relationships between the immune system and diseases. Int J Mol Sci. 2022;23(7):3636. 35408994 10.3390/ijms23073636 PMC8998851

[50] Baima G , Minoli M , Michaud DS , et al. Periodontitis and risk of cancer: mechanistic evidence. Periodontol 2000. 2024;96:83‐94. 38102837 10.1111/prd.12540 PMC11579815

[51] Armitage GC . Clinical evaluation of periodontal diseases. Periodontol 2000. 2000;1995(7):39‐53. 10.1111/j.1600-0757.1995.tb00035.x 9567929

[52] Papapanou PN , Sanz M , Buduneli N , et al. Periodontitis: consensus report of workgroup 2 of the 2017 world workshop on the classification of periodontal and Peri‐implant diseases and conditions. J Clin Periodontol. 2018;45(Suppl 20):S162‐S170. 29926490 10.1111/jcpe.12946

[53] Loos BG , Craandijk J , Hoek FJ , Wertheim‐van Dillen PM , van der Velden U . Elevation of systemic markers related to cardiovascular diseases in the peripheral blood of periodontitis patients. J Periodontol. 2000;71(10):1528‐1534. 11063384 10.1902/jop.2000.71.10.1528

[54] Botelho J , Machado V , Hussain SB , et al. Periodontitis and circulating blood cell profiles: a systematic review and meta‐analysis. Exp Hematol. 2021;93:1‐13. 33068648 10.1016/j.exphem.2020.10.001

[55] Gong S , Gao X , Xu F , et al. Association of lymphocyte to monocyte ratio with severity of coronary artery disease. Medicine (Baltimore). 2018;97(43):e12813. 30412071 10.1097/MD.0000000000012813 PMC6221743

[56] Jenne CN , Liao S , Singh B . Neutrophils: multitasking first responders of immunity and tissue homeostasis. Cell Tissue Res. 2018;371(3):395‐397. 29392468 10.1007/s00441-018-2802-5

[57] Schroeder HE , Münzel‐Pedrazzoli S , Page R . Correlated morphometric and biochemical analysis of gingival tissue in early chronic gingivitis in man. Arch Oral Biol. 1973;18(7):899‐923. 4516188 10.1016/0003-9969(73)90060-5

[58] Schiött CR , Löe H . The origin and variation in number of leukocytes in the human saliva. J Periodontal Res. 1970;5(1):36‐41. 4255144 10.1111/j.1600-0765.1970.tb01835.x

[59] Vitkov L , Muñoz LE , Knopf J , et al. Connection between periodontitis‐induced low‐grade Endotoxemia and systemic diseases: neutrophils as protagonists and targets. Int J Mol Sci. 2021;22(9):4647. 33925019 10.3390/ijms22094647 PMC8125370

[60] Chapple ILC , Hirschfeld J , Kantarci A , Wilensky A , Shapira L . The role of the host‐neutrophil biology. Periodontol 2000. 2023. 10.1111/prd.12490 37199393

[61] Van Dyke TE , Hoop GA . Neutrophil function and oral disease. Crit Rev Oral Biol Med. 1990;1(2):117‐133. 2152247 10.1177/10454411900010020201

[62] Brinkmann V , Reichard U , Goosmann C , et al. Neutrophil extracellular traps kill bacteria. Science. 2004;303(5663):1532‐1535. 15001782 10.1126/science.1092385

[63] White PC , Chicca IJ , Cooper PR , Milward MR , Chapple IL . Neutrophil extracellular traps in periodontitis: a web of intrigue. J Dent Res. 2016;95(1):26‐34. 26442948 10.1177/0022034515609097

[64] Khoury W , Glogauer J , Tenenbaum HC , Glogauer M . Oral inflammatory load: neutrophils as oral health biomarkers. J Periodontal Res. 2020;55(5):594‐601. 32372438 10.1111/jre.12758

[65] Groeger S , Meyle J . Oral mucosal epithelial cells. Front Immunol. 2019;10:208. 30837987 10.3389/fimmu.2019.00208 PMC6383680

[66] Sochalska M , Potempa J . Manipulation of neutrophils by *Porphyromonas gingivalis* in the development of periodontitis. Front Cell Infect Microbiol. 2017;7:197. 28589098 10.3389/fcimb.2017.00197 PMC5440471

[67] Anand PS , Sagar DK , Mishra S , Narang S , Kamath KP , Anil S . Total and differential leukocyte counts in the peripheral blood of patients with generalised aggressive periodontitis. Oral Health Prev Dent. 2016;14(5):443‐450. 27351735 10.3290/j.ohpd.a36470

[68] Christan C , Dietrich T , Hägewald S , Kage A , Bernimoulin JP . White blood cell count in generalized aggressive periodontitis after non‐surgical therapy. J Clin Periodontol. 2002;29(3):201‐206. 11940137 10.1034/j.1600-051x.2002.290303.x

[69] Laki K . Our ancient heritage in blood clotting and some of its consequences. Ann N Y Acad Sci. 1972;202:297‐307. 4508929 10.1111/j.1749-6632.1972.tb16342.x

[70] Machlus KR , Thon JN , Italiano JE Jr . Interpreting the developmental dance of the megakaryocyte: a review of the cellular and molecular processes mediating platelet formation. Br J Haematol. 2014;165(2):227‐236. 24499183 10.1111/bjh.12758

[71] Vinholt PJ , Hvas AM , Frederiksen H , Bathum L , Jørgensen MK , Nybo M . Platelet count is associated with cardiovascular disease, cancer and mortality: a population‐based cohort study. Thromb Res. 2016;148:136‐142. 27586589 10.1016/j.thromres.2016.08.012

[72] Kabat GC , Kim MY , Verma AK , et al. Platelet count and total and cause‐specific mortality in the Women's Health Initiative. Ann Epidemiol. 2017;27(4):274‐280. 28320576 10.1016/j.annepidem.2017.02.001

[73] Thaulow E , Erikssen J , Sandvik L , Stormorken H , Cohn PF . Blood platelet count and function are related to total and cardiovascular death in apparently healthy men. Circulation. 1991;84(2):613‐617. 1860204 10.1161/01.cir.84.2.613

[74] D'Aiuto F , Parkar M , Andreou G , et al. Periodontitis and systemic inflammation: control of the local infection is associated with a reduction in serum inflammatory markers. J Dent Res. 2004;83(2):156‐160. 14742655 10.1177/154405910408300214

[75] Hsu HC , Tsai WH , Jiang ML , et al. Circulating levels of thrombopoietic and inflammatory cytokines in patients with clonal and reactive thrombocytosis. J Lab Clin Med. 1999;134(4):392‐397. 10521086 10.1016/s0022-2143(99)90154-3

[76] Kaser A , Brandacher G , Steurer W , et al. Interleukin‐6 stimulates thrombopoiesis through thrombopoietin: role in inflammatory thrombocytosis. Blood. 2001;98(9):2720‐2725. 11675343 10.1182/blood.v98.9.2720

[77] Kaushansky K . Determinants of platelet number and regulation of thrombopoiesis. Hematology Am Soc Hematol Educ Program. 2009;147‐152. 20008193 10.1182/asheducation-2009.1.147

[78] Kapur R , Zufferey A , Boilard E , Semple JW . Nouvelle cuisine: platelets served with inflammation. J Immunol. 2015;194(12):5579‐5587. 26048965 10.4049/jimmunol.1500259

[79] Semple JW , Italiano JE Jr , Freedman J . Platelets and the immune continuum. Nat Rev Immunol. 2011;11(4):264‐274. 21436837 10.1038/nri2956

[80] Bakogiannis C , Sachse M , Stamatelopoulos K , Stellos K . Platelet‐derived chemokines in inflammation and atherosclerosis. Cytokine. 2019;122:154157. 29198385 10.1016/j.cyto.2017.09.013

[81] Herzberg MC , Meyer MW . Effects of oral flora on platelets: possible consequences in cardiovascular disease. J Periodontol. 1996;67(10 Suppl):1138‐1142. 10.1902/jop.1996.67.10s.1138 8910832

[82] Monteiro AM , Jardini MA , Alves S , et al. Cardiovascular disease parameters in periodontitis. J Periodontol. 2009;80(3):378‐388. 19254121 10.1902/jop.2009.080431

[83] Gaertner F , Ahmad Z , Rosenberger G , et al. Migrating platelets are Mechano‐scavengers that collect and bundle bacteria. Cell. 2017;171(6):1368‐1382.e3. 29195076 10.1016/j.cell.2017.11.001

[84] Gawaz M , Vogel S . Platelets in tissue repair: control of apoptosis and interactions with regenerative cells. Blood. 2013;122(15):2550‐2554. 23963043 10.1182/blood-2013-05-468694

[85] Brousseau‐Nault M , Kizhakkedathu JN , Kim H . Chronic periodontitis is associated with platelet factor 4 (PF4) secretion: a pilot study. J Clin Periodontol. 2017;44(11):1101‐1111. 28681377 10.1111/jcpe.12771

[86] Nicu EA , Van der Velden U , Nieuwland R , Everts V , Loos BG . Elevated platelet and leukocyte response to oral bacteria in periodontitis. J Thromb Haemost. 2009;7(1):162‐170. 18983491 10.1111/j.1538-7836.2008.03219.x

[87] Al‐Rasheed A . Elevation of white blood cells and platelet counts in patients having chronic periodontitis. Saudi Dent J. 2012;24(1):17‐21. 23960523 10.1016/j.sdentj.2011.10.006 PMC3723072

[88] Kumar BP , Khaitan T , Ramaswamy P , Sreenivasulu P , Uday G , Velugubantla RG . Association of chronic periodontitis with white blood cell and platelet count – a case control study. J Clin Exp Dent. 2014;6(3):e214‐e217. 25136419 10.4317/jced.51292 PMC4134847

[89] Romandini M , Laforí A , Romandini P , Baima G , Cordaro M . Periodontitis and platelet count: a new potential link with cardiovascular and other systemic inflammatory diseases. J Clin Periodontol. 2018;45(11):1299‐1310. 30133784 10.1111/jcpe.13004

[90] Acharya AB , Shetty IP , Jain S , et al. Neutrophil‐to‐lymphocyte ratio and platelet‐to‐lymphocyte ratio in chronic periodontitis before and after nonsurgical therapy. J Indian Soc Periodontol. 2019;23(5):419‐423. 31543614 10.4103/jisp.jisp_622_18 PMC6737853

[91] Lu R , Li W , Wang X , Shi D , Meng H . Elevated neutrophil‐to‐lymphocyte ratio but not platelet‐to‐lymphocyte ratio is associated with generalized aggressive periodontitis in a Chinese population. J Periodontol. 2021;92(4):507‐513. 32909291 10.1002/JPER.20-0282

[92] Papapanagiotou D , Nicu EA , Bizzarro S , et al. Periodontitis is associated with platelet activation. Atherosclerosis. 2009;202(2):605‐611. 18617175 10.1016/j.atherosclerosis.2008.05.035

[93] Arvanitidis E , Bizzarro S , Alvarez Rodriguez E , Loos BG , Nicu EA . Reduced platelet hyper‐reactivity and platelet‐leukocyte aggregation after periodontal therapy. Thromb J. 2017;15:5. 28190975 10.1186/s12959-016-0125-x PMC5292810

[94] Laky M , Anscheringer I , Wolschner L , et al. Periodontal treatment limits platelet activation in patients with periodontitis‐a controlled‐randomized intervention trial. J Clin Periodontol. 2018;45(9):1090‐1097. 29972709 10.1111/jcpe.12980

[95] Zhan Y , Lu R , Meng H , et al. Platelets as inflammatory mediators in a murine model of periodontitis. J Clin Periodontol. 2020;47(5):572‐582. 32017185 10.1111/jcpe.13265

[96] Shenker BJ , Datar S . Fusobacterium nucleatum inhibits human T‐cell activation by arresting cells in the mid‐G1 phase of the cell cycle. Infect Immun. 1995;63(12):4830‐4836. 7591143 10.1128/iai.63.12.4830-4836.1995 PMC173692

[97] Stashenko P , Resmini LM , Haffajee AD , Socransky SS . T cell responses of periodontal disease patients and healthy subjects to oral microorganisms. J Periodontal Res. 1983;18(6):587‐600. 6230432 10.1111/j.1600-0765.1983.tb00396.x

[98] Jankovic D , Feng CG . CD4(+) T cell differentiation in infection: amendments to the Th1/Th2 axiom. Front Immunol. 2015;6:198. 25972870 10.3389/fimmu.2015.00198 PMC4413827

[99] Aoyagi T , Sugawara‐Aoyagi M , Yamazaki K , Hara K . Interleukin 4 (IL‐4) and IL‐6‐producing memory T‐cells in peripheral blood and gingival tissue in periodontitis patients with high serum antibody titers to *Porphyromonas gingivalis* . Oral Microbiol Immunol. 1995;10(5):304‐310. 8596674 10.1111/j.1399-302x.1995.tb00159.x

[100] Tokoro Y , Matsuki Y , Yamamoto T , Suzuki T , Hara K . Relevance of local Th2‐type cytokine mRNA expression in immunocompetent infiltrates in inflamed gingival tissue to periodontal diseases. Clin Exp Immunol. 1997;107(1):166‐174. 9010272 10.1046/j.1365-2249.1997.d01-880.x PMC1904550

[101] Wassenaar A , Reinhardus C , Thepen T , Abraham‐Inpijn L , Kievits F . Cloning, characterization, and antigen specificity of T‐lymphocyte subsets extracted from gingival tissue of chronic adult periodontitis patients. Infect Immun. 1995;63(6):2147‐2153. 7539406 10.1128/iai.63.6.2147-2153.1995 PMC173279

[102] Takeichi O , Haber J , Kawai T , Smith DJ , Moro I , Taubman MA . Cytokine profiles of T‐lymphocytes from gingival tissues with pathological pocketing. J Dent Res. 2000;79(8):1548‐1555. 11023273 10.1177/00220345000790080401

[103] Ukai T , Mori Y , Onoyama M , Hara Y . Immunohistological study of interferon‐gamma‐ and interleukin‐4‐bearing cells in human periodontitis gingiva. Arch Oral Biol. 2001;46(10):901‐908. 11451404 10.1016/s0003-9969(01)00057-7

[104] Berglundh T , Liljenberg B , Lindhe J . Some cytokine profiles of T‐helper cells in lesions of advanced periodontitis. J Clin Periodontol. 2002;29(8):705‐709. 12390567 10.1034/j.1600-051x.2002.290807.x

[105] O'Connor W Jr , Zenewicz LA , Flavell RA . The dual nature of T(H)17 cells: shifting the focus to function. Nat Immunol. 2010;11(6):471‐476. 20485275 10.1038/ni.1882

[106] Singh RP , Hasan S , Sharma S , et al. Th17 cells in inflammation and autoimmunity. Autoimmun Rev. 2014;13(12):1174‐1181. 25151974 10.1016/j.autrev.2014.08.019

[107] Stadhouders R , Lubberts E , Hendriks RW . A cellular and molecular view of T helper 17 cell plasticity in autoimmunity. J Autoimmun. 2018;87:1‐15. 29275836 10.1016/j.jaut.2017.12.007

[108] van Bruggen N , Ouyang W . Th17 cells at the crossroads of autoimmunity, inflammation, and atherosclerosis. Immunity. 2014;40(1):10‐12. 24439264 10.1016/j.immuni.2013.12.006

[109] Adibrad M , Deyhimi P , Ganjalikhani Hakemi M , Behfarnia P , Shahabuei M , Rafiee L . Signs of the presence of Th17 cells in chronic periodontal disease. J Periodontal Res. 2012;47(4):525‐531. 22309127 10.1111/j.1600-0765.2011.01464.x

[110] Okui T , Aoki Y , Ito H , Honda T , Yamazaki K . The presence of IL‐17+/FOXP3+ double‐positive cells in periodontitis. J Dent Res. 2012;91(6):574‐579. 22522772 10.1177/0022034512446341

[111] Jia R , Hashizume‐Takizawa T , Du Y , Yamamoto M , Kurita‐Ochiai T . Aggregatibacter actinomycetemcomitans induces Th17 cells in atherosclerotic lesions. Pathog Dis. 2015;73(3):ftu027. 25743474 10.1093/femspd/ftu027

[112] Sharara SL , Tayyar R , Kanafani ZA , Kanj SS . HACEK endocarditis: a review. Expert Rev Anti Infect Ther. 2016;14(6):539‐545. 27124204 10.1080/14787210.2016.1184085

[113] Candelli M , Franza L , Pignataro G , et al. Interaction between lipopolysaccharide and gut microbiota in inflammatory bowel diseases. Int J Mol Sci. 2021;22(12):6242. 34200555 10.3390/ijms22126242 PMC8226948

[114] Yang J , Zhao Y , Shao F . Non‐canonical activation of inflammatory caspases by cytosolic LPS in innate immunity. Curr Opin Immunol. 2015;32:78‐83. 25621708 10.1016/j.coi.2015.01.007

[115] Heinbockel L , Weindl G , Martinez‐de‐Tejada G , et al. Inhibition of lipopolysaccharide‐ and lipoprotein‐induced inflammation by antitoxin peptide Pep19‐2.5. Front Immunol. 2018;9:1704. 30093904 10.3389/fimmu.2018.01704 PMC6070603

[116] Zhang L , Gao L , Xu C , et al. Porphyromonas gingivalis lipopolysaccharide promotes T‐ helper 17 cell differentiation from human CD4(+) naïve T cells via toll‐like receptor‐2 in vitro. Arch Oral Biol. 2019;107:104483. 31351339 10.1016/j.archoralbio.2019.104483

[117] Chaudhry A , Rudensky AY . Control of inflammation by integration of environmental cues by regulatory T cells. J Clin Invest. 2013;123(3):939‐944. 23454755 10.1172/JCI57175 PMC3582113

[118] Shevach EM . Foxp3(+) T regulatory cells: still many unanswered questions‐a perspective after 20 years of study. Front Immunol. 2018;9:1048. 29868011 10.3389/fimmu.2018.01048 PMC5962663

[119] Cardoso CR , Garlet GP , Moreira AP , Júnior WM , Rossi MA , Silva JS . Characterization of CD4+CD25+ natural regulatory T cells in the inflammatory infiltrate of human chronic periodontitis. J Leukoc Biol. 2008;84(1):311‐318. 18451325 10.1189/jlb.0108014

[120] Dutzan N , Gamonal J , Silva A , Sanz M , Vernal R . Over‐expression of forkhead box P3 and its association with receptor activator of nuclear factor‐kappa B ligand, interleukin (IL) ‐17, IL‐10 and transforming growth factor‐beta during the progression of chronic periodontitis. J Clin Periodontol. 2009;36(5):396‐403. 19419438 10.1111/j.1600-051X.2009.01390.x

[121] Nakajima T , Ueki‐Maruyama K , Oda T , et al. Regulatory T‐cells infiltrate periodontal disease tissues. J Dent Res. 2005;84(7):639‐643. 15972593 10.1177/154405910508400711

[122] Ernst CW , Lee JE , Nakanishi T , et al. Diminished forkhead box P3/CD25 double‐positive T regulatory cells are associated with the increased nuclear factor‐kappaB ligand (RANKL+) T cells in bone resorption lesion of periodontal disease. Clin Exp Immunol. 2007;148(2):271‐280. 17355249 10.1111/j.1365-2249.2006.03318.x PMC1868884

[123] Nielsen MM , Witherden DA , Havran WL . γδ T cells in homeostasis and host defence of epithelial barrier tissues. Nat Rev Immunol. 2017;17(12):733‐745. 28920588 10.1038/nri.2017.101 PMC5771804

[124] Chitadze G , Oberg HH , Wesch D , Kabelitz D . The ambiguous role of γδ T lymphocytes in antitumor immunity. Trends Immunol. 2017;38(9):668‐678. 28709825 10.1016/j.it.2017.06.004

[125] Silva‐Santos B , Mensurado S , Coffelt SB . γδ T cells: pleiotropic immune effectors with therapeutic potential in cancer. Nat Rev Cancer. 2019;19(7):392‐404. 31209264 10.1038/s41568-019-0153-5 PMC7614706

[126] Coffelt SB , Kersten K , Doornebal CW , et al. IL‐17‐producing γδ T cells and neutrophils conspire to promote breast cancer metastasis. Nature. 2015;522(7556):345‐348. 25822788 10.1038/nature14282 PMC4475637

[127] Ribot JC , Ribeiro ST , Correia DV , Sousa AE , Silva‐Santos B . Human γδ thymocytes are functionally immature and differentiate into cytotoxic type 1 effector T cells upon IL‐2/IL‐15 signaling. J Immunol. 2014;192(5):2237‐2243. 24489097 10.4049/jimmunol.1303119

[128] Hovav AH , Wilensky A . The role of the epithelial sentinels, Langerhans cells and γδT cells, in oral squamous cell carcinoma. Periodontol 2000. 2024;96:221‐228. 38273461 10.1111/prd.12544 PMC11579810

[129] Xu W , Zhou W , Wang H , Liang S . Roles of *Porphyromonas gingivalis* and its virulence factors in periodontitis. Adv Protein Chem Struct Biol. 2020;120:45‐84. 32085888 10.1016/bs.apcsb.2019.12.001 PMC8204362

[130] Lindhe J , Ranney R , Lamster I , et al. Consensus report: chronic periodontitis. Ann Periodontol. 1999;4(1):38.

[131] Bhattacharya HS , Srivastava R , Gummaluri SS , Agarwal MC , Bhattacharya P , Astekar MS . Comparison of blood parameters between periodontitis patients and healthy participants: a cross‐sectional hematological study. J Oral Maxillofac Pathol. 2022;26(1):77‐81. 35571313 10.4103/jomfp.jomfp_349_21 PMC9106243

[132] Caton JG , Armitage G , Berglundh T , et al. A new classification scheme for periodontal and peri‐implant diseases and conditions – introduction and key changes from the 1999 classification. J Clin Periodontol. 2018;45(Suppl 20):S1‐S8. 29926489 10.1111/jcpe.12935

[133] Çetin Özdemir E , Bilen E , Yazar FM . Can the delta neutrophil ındex be used as a preliminary biomarker ın the evaluation of periodontal disease: a pilot study. J Appl Oral Sci. 2022;30:e20210555. 35319605 10.1590/1678-7757-2021-0555 PMC8963392

[134] Chapple ILC , Mealey BL , Van Dyke TE , et al. Periodontal health and gingival diseases and conditions on an intact and a reduced periodontium: consensus report of workgroup 1 of the 2017 World Workshop on the classification of periodontal and peri‐implant diseases and conditions. J Clin Periodontol. 2018;45(Suppl 20):S68‐S77. 29926499 10.1111/jcpe.12940

[135] Doğan B , Fentoğlu Ö , Kırzıoğlu FY , et al. Lipoxin A4 and neutrophil/lymphocyte ratio: a possible indicator in achieved systemic risk factors for periodontitis. Med Sci Monit. 2015;21:2485‐2493. 26298769 10.12659/MSM.895115 PMC4551304

[136] Armitage GC . Development of a classification system for periodontal diseases and conditions. Ann Periodontol. 1999;4(1):1‐6. 10863370 10.1902/annals.1999.4.1.1

[137] Mishra S , Johnson L , Gazala MP , Dahiya S , Rahman W , Sreeraj VS . Systemic immune‐inflammation index in patients with generalized stage III grade C periodontitis. Oral Dis. 2022;29:3599‐3609. 35913425 10.1111/odi.14328

[138] Mishra S , Gazala MP , Rahman W . Clinical and diagnostic significance of blood leukocyte ratios in young patients with stage III grade C periodontitis. Acta Odontol Scand. 2022;80(3):161‐168. 34436974 10.1080/00016357.2021.1969035

[139] Temelli B , Yetkin Ay Z , Aksoy F , et al. Platelet indices (mean platelet volume and platelet distribution width) have correlations with periodontal inflamed surface area in coronary artery disease patients: a pilot study. J Periodontol. 2018;89(10):1203‐1212. 29802642 10.1002/JPER.17-0684

[140] Mariotti A . Dental plaque‐induced gingival diseases. Ann Periodontol. 1999;4(1):7‐19. 10863371 10.1902/annals.1999.4.1.7

[141] Torrungruang K , Ongphiphadhanakul B , Jitpakdeebordin S , Sarujikumjornwatana S . Mediation analysis of systemic inflammation on the association between periodontitis and glycaemic status. J Clin Periodontol. 2018;45(5):548‐556. 29500831 10.1111/jcpe.12884

[142] American‐Diabetes‐Association . Diagnosis and classification of diabetes mellitus. Diabetes Care. 2014;37(Suppl 1):S81‐S90. 24357215 10.2337/dc14-S081

[143] Page RC , Eke PI . Case definitions for use in population‐based surveillance of periodontitis. J Periodontol. 2007;78(7 Suppl):1387‐1399. 10.1902/jop.2007.060264 17608611

[144] Mutthineni RB , Ramishetty A , Gojja P , Muralidaran G , Burle VVA . Platelet indices be a new biomarker for periodontal disease. Contemp Clin Dent. 2021;12(3):289‐293. 34759687 10.4103/ccd.ccd_461_20 PMC8525805

[145] Ustaoglu G , Erdal E , İnanır M . Does periodontitis affect mean platelet volume (MPV) and plateletcrit (PCT) levels in healthy adults? Rev Assoc Med Bras. 2020;66(2):133‐138. 32428146 10.1590/1806-9282.66.2.133

[146] Anand PS , Sagar DK , Ashok S , Kamath KP . Association of aggressive periodontitis with reduced erythrocyte counts and reduced hemoglobin levels. J Periodontal Res. 2014;49(6):719‐728. 24329044 10.1111/jre.12154

[147] López R , Loos BG , Baelum V . Hematological features in adolescents with periodontitis. Clin Oral Investig. 2012;16(4):1209‐1216. 10.1007/s00784-011-0628-6 22009185

[148] Sridharan S , Sravani P , Rao RJ . Coefficient of variation of red cell distribution width has correlations to periodontal inflamed surface area in non‐obese hypertensive patients. J Int Acad Periodontol. 2021;23(2):106‐114. 33929811

[149] Cao R , Li C , Geng F , Pan Y . J‐shaped association between systemic immune‐inflammation index and periodontitis: results from NHANES 2009‐2014. J Periodontol. 2024;95(4):397‐406. 37713193 10.1002/JPER.23-0260

[150] Hajishengallis G . Interconnection of periodontal disease and comorbidities: evidence, mechanisms, and implications. Periodontol 2000. 2022;89(1):9‐18. 35244969 10.1111/prd.12430 PMC9018559

[151] Azab B , Bhatt VR , Phookan J , et al. Usefulness of the neutrophil‐to‐lymphocyte ratio in predicting short‐ and long‐term mortality in breast cancer patients. Ann Surg Oncol. 2012;19(1):217‐224. 21638095 10.1245/s10434-011-1814-0

[152] Azab B , Camacho‐Rivera M , Taioli E . Average values and racial differences of neutrophil lymphocyte ratio among a nationally representative sample of United States subjects. PLoS One. 2014;9(11):e112361. 25375150 10.1371/journal.pone.0112361 PMC4223021

[153] Lee JS , Kim NY , Na SH , Youn YH , Shin CS . Reference values of neutrophil‐lymphocyte ratio, lymphocyte‐monocyte ratio, platelet‐lymphocyte ratio, and mean platelet volume in healthy adults in South Korea. Medicine (Baltimore). 2018;97(26):e11138. 29952958 10.1097/MD.0000000000011138 PMC6039688

[154] Ferguson JF , Patel PN , Shah RY , et al. Race and gender variation in response to evoked inflammation. J Transl Med. 2013;11:63. 23497455 10.1186/1479-5876-11-63 PMC3636014

[155] Oh BS , Jang JW , Kwon JH , et al. Prognostic value of C‐reactive protein and neutrophil‐to‐lymphocyte ratio in patients with hepatocellular carcinoma. BMC Cancer. 2013;13:78. 23409924 10.1186/1471-2407-13-78 PMC3584844

[156] Isaac V , Wu CY , Huang CT , Baune BT , Tseng CL , McLachlan CS . Elevated neutrophil to lymphocyte ratio predicts mortality in medical inpatients with multiple chronic conditions. Medicine (Baltimore). 2016;95(23):e3832. 27281085 10.1097/MD.0000000000003832 PMC4907663

[157] Rha MS , Kim CH , Yoon JH , Cho HJ . Association between the neutrophil‐to‐lymphocyte ratio and obstructive sleep apnea: a meta‐analysis. Sci Rep. 2020;10(1):10862. 32616762 10.1038/s41598-020-67708-w PMC7331605

[158] Tonyali S , Ceylan C , Yahsi S , Karakan MS . Does neutrophil to lymphocyte ratio demonstrate deterioration in renal function? Ren Fail. 2018;40(1):209‐212. 29616601 10.1080/0886022X.2018.1455590 PMC6014370

[159] Cupp MA , Cariolou M , Tzoulaki I , Aune D , Evangelou E , Berlanga‐Taylor AJ . Neutrophil to lymphocyte ratio and cancer prognosis: an umbrella review of systematic reviews and meta‐analyses of observational studies. BMC Med. 2020;18(1):360. 33213430 10.1186/s12916-020-01817-1 PMC7678319

[160] Guo X , Zhang S , Zhang Q , et al. Neutrophil:lymphocyte ratio is positively related to type 2 diabetes in a large‐scale adult population: a Tianjin chronic low‐grade systemic inflammation and health cohort study. Eur J Endocrinol. 2015;173(2):217‐225. 25953830 10.1530/EJE-15-0176

[161] Herrmann JM , Sonnenschein SK , Groeger SE , Ewald N , Arneth B , Meyle J . Refractory neutrophil activation in type 2 diabetics with chronic periodontitis. J Periodontal Res. 2020;55(2):315‐323. 31912903 10.1111/jre.12717

[162] Noack B , Jachmann I , Roscher S , et al. Metabolic diseases and their possible link to risk indicators of periodontitis. J Periodontol. 2000;71(6):898‐903. 10914792 10.1902/jop.2000.71.6.898

[163] Fentoğlu O , Oz G , Taşdelen P , Uskun E , Aykaç Y , Bozkurt FY . Periodontal status in subjects with hyperlipidemia. J Periodontol. 2009;80(2):267‐273. 19186967 10.1902/jop.2009.080104

[164] Fentoglu O , Bozkurt FY . The Bi‐directional relationship between periodontal disease and hyperlipidemia. Eur J Dent. 2008;2(2):142‐146. 19212526 PMC2633171

[165] Lin BD , Hottenga JJ , Abdellaoui A , et al. Causes of variation in the neutrophil‐lymphocyte and platelet‐lymphocyte ratios: a twin‐family study. Biomark Med. 2016;10(10):1061‐1072. 27690543 10.2217/bmm-2016-0147 PMC5220440

[166] Umehara T , Oka H , Nakahara A , Matsuno H , Murakami H . Differential leukocyte count is associated with clinical phenotype in Parkinson's disease. J Neurol Sci. 2020;409:116638. 31865186 10.1016/j.jns.2019.116638

[167] Rhee H , Love T , Harrington D . Blood neutrophil count is associated with body mass index in adolescents with asthma. JSM Allergy Asthma. 2018;3(1):1019. 30542672 PMC6287916

[168] Furuncuoğlu Y , Tulgar S , Dogan AN , Cakar S , Tulgar YK , Cakiroglu B . How obesity affects the neutrophil/lymphocyte and platelet/lymphocyte ratio, systemic immune‐inflammatory index and platelet indices: a retrospective study. Eur Rev Med Pharmacol Sci. 2016;20(7):1300‐1306. 27097950

[169] Zhan Y , Lu R , Meng H , Wang X , Sun X , Hou J . The role of platelets in inflammatory immune responses in generalized aggressive periodontitis. J Clin Periodontol. 2017;44(2):150‐157. 27883202 10.1111/jcpe.12657

[170] Nibali L , Darbar U , Rakmanee T , Donos N . Anemia of inflammation associated with periodontitis: analysis of two clinical studies. J Periodontol. 2019;90(11):1252‐1259. 31119743 10.1002/JPER.19-0124

[171] Zhan Y , Lu R , Meng H , Wang X , Hou J . Platelet activation and platelet‐leukocyte interaction in generalized aggressive periodontitis. J Leukoc Biol. 2016;100(5):1155‐1166. 27334227 10.1189/jlb.4A1115-526RR

[172] Gaddale R , Mudda JA , Karthikeyan I , Desai SR , Shinde H , Deshpande P . Changes in cellular and molecular components of peripheral blood in patients with generalized aggressive periodontitis. J Investig Clin Dent. 2016;7(1):59‐64. 10.1111/jicd.12127 25283691

[173] Almășan O , Leucuța DC , Hedeșiu M . Blood cell count inflammatory markers as prognostic indicators of periodontitis: a systematic review and meta‐analysis. J Pers Med. 2022;12(6):992. 35743775 10.3390/jpm12060992 PMC9225277

[174] Ahn C , Kim W , Lim TH , Cho Y , Choi KS , Jang BH . The delta neutrophil index (DNI) as a prognostic marker for mortality in adults with sepsis: a systematic review and meta‐analysis. Sci Rep. 2018;8(1):6621. 29700315 10.1038/s41598-018-24211-7 PMC5919925

[175] Kim TY , Kim SJ , Kim YS , et al. Delta neutrophil index as an early predictive marker of severe acute pancreatitis in the emergency department. United European Gastroenterol J. 2019;7(4):488‐495. 10.1177/2050640619838359 PMC6488799 31065366

[176] Nahm CH , Choi JW , Lee J . Delta neutrophil index in automated immature granulocyte counts for assessing disease severity of patients with sepsis. Ann Clin Lab Sci. 2008;38(3):241‐246. 18715852

[177] Park JH , Byeon HJ , Lee KH , et al. Delta neutrophil index (DNI) as a novel diagnostic and prognostic marker of infection: a systematic review and meta‐analysis. Inflamm Res. 2017;66(10):863‐870. 28646289 10.1007/s00011-017-1066-y

[178] Ahn JG , Choi SY , Kim DS , Kim KH . Limitation of the delta neutrophil index for assessing bacteraemia in immunocompromised children. Clin Chim Acta. 2014;436:319‐322. 24978822 10.1016/j.cca.2014.06.020

[179] Han SI , Cha KC , Roh YI , Hwang SO , Jung WJ , Kim TY . Association between novel marker (platelet‐lymphocyte ratio, neutrophil‐lymphocyte ratio, and Delta neutrophil index) and outcomes in sudden cardiac arrest patients. Emerg Med Int. 2021;2021:6650958. 33833877 10.1155/2021/6650958 PMC8012140

[180] Birben B , Birben OD , Akın T , et al. Efficacy of the delta neutrophil index in predicting 30‐day mortality in COVID‐19 patients requiring intensive care. Int J Clin Pract. 2021;75(5):e13970. 33368905 10.1111/ijcp.13970 PMC7883061

[181] Ko DR , Jang JE , Chung SP , et al. Usefulness of the delta neutrophil index as an ancillary test in the emergency department for the early diagnosis of suspected acute promyelocytic leukemia. Leuk Lymphoma. 2017;58(10):2387‐2394. 28278698 10.1080/10428194.2017.1296142

[182] Bozan MB , Yazar FM , Kale İT , Yüzbaşıoğlu MF , Boran ÖF , Azak Bozan A . Delta neutrophil index and neutrophil‐to‐lymphocyte ratio in the differentiation of thyroid malignancy and nodular goiter. World J Surg. 2021;45(2):507‐514. 33067685 10.1007/s00268-020-05822-6

[183] Bozan MB , Yazar FM , Kale IT , Topuz S , Bozan AA , Boran OF . Immature granulocyte count and Delta neutrophil index as new predictive factors for axillary metastasis of breast cancer. J Coll Physicians Surg Pak. 2022;32(2):220‐225. 35108795 10.29271/jcpsp.2022.02.220

[184] Barut O , Demirkol MK , Kucukdurmaz F , Sahinkanat T , Resim S . Pre‐treatment Delta neutrophil index as a predictive factor in renal cell carcinoma. J Coll Physicians Surg Pak. 2021;31(2):156‐161. 33645181 10.29271/jcpsp.2021.02.156

[185] Wang J , Zhang F , Jiang F , Hu L , Chen J , Wang Y . Distribution and reference interval establishment of neutral‐to‐lymphocyte ratio (NLR), lymphocyte‐to‐monocyte ratio (LMR), and platelet‐to‐lymphocyte ratio (PLR) in Chinese healthy adults. J Clin Lab Anal. 2021;35(9):e23935. 34390017 10.1002/jcla.23935 PMC8418511

[186] Du J , Chen S , Shi J , et al. The association between the lymphocyte‐monocyte ratio and disease activity in rheumatoid arthritis. Clin Rheumatol. 2017;36(12):2689‐2695. 28913574 10.1007/s10067-017-3815-2

[187] Lin S , Fang Y , Mo Z , Lin Y , Ji C , Jian Z . Prognostic value of lymphocyte to monocyte ratio in pancreatic cancer: a systematic review and meta‐analysis including 3338 patients. World J Surg Oncol. 2020;18(1):186. 32711514 10.1186/s12957-020-01962-0 PMC7382838

[188] Tan D , Fu Y , Tong W , Li F . Prognostic significance of lymphocyte to monocyte ratio in colorectal cancer: a meta‐analysis. Int J Surg. 2018;55:128‐138. 29807167 10.1016/j.ijsu.2018.05.030

[189] Wang Y , Xu C , Zhang Z . Prognostic value of pretreatment lymphocyte‐to‐monocyte ratio in patients with glioma: a meta‐analysis. BMC Med. 2023;21(1):486. 38053096 10.1186/s12916-023-03199-6 PMC10696791

[190] Jin J , Yang L , Liu D , Li WM . Prognostic value of pretreatment lymphocyte‐to‐monocyte ratio in lung cancer: a systematic review and meta‐analysis. Technol Cancer Res Treat. 2021;20:1533033820983085. 33576324 10.1177/1533033820983085 PMC7887688

[191] Hu G , Liu G , Ma JY , Hu RJ . Lymphocyte‐to‐monocyte ratio in esophageal squamous cell carcinoma prognosis. Clin Chim Acta. 2018;486:44‐48. 30028962 10.1016/j.cca.2018.07.029

[192] Ma JY , Liu Q . Clinicopathological and prognostic significance of lymphocyte to monocyte ratio in patients with gastric cancer: a meta‐analysis. Int J Surg. 2018;50:67‐71. 29329786 10.1016/j.ijsu.2018.01.002

[193] Hamid HKS , Emile SH , Davis GN . Prognostic significance of lymphocyte‐to‐monocyte and platelet‐to‐lymphocyte ratio in rectal cancer: a systematic review, meta‐analysis, and meta‐regression. Dis Colon Rectum. 2022;65(2):178‐187. 34775400 10.1097/DCR.0000000000002291

[194] Gu L , Li H , Chen L , et al. Prognostic role of lymphocyte to monocyte ratio for patients with cancer: evidence from a systematic review and meta‐analysis. Oncotarget. 2016;7(22):31926‐31942. 26942464 10.18632/oncotarget.7876 PMC5077986

[195] Wei D , Liu J , Ma J . The value of lymphocyte to monocyte ratio in the prognosis of head and neck squamous cell carcinoma: a meta‐analysis. PeerJ. 2023;11:e16014. 37719125 10.7717/peerj.16014 PMC10501369

[196] Li B , Zhou P , Liu Y , et al. Platelet‐to‐lymphocyte ratio in advanced cancer: review and meta‐analysis. Clin Chim Acta. 2018;483:48‐56. 29678631 10.1016/j.cca.2018.04.023

[197] Cao W , Yu H , Zhu S , et al. Clinical significance of preoperative neutrophil‐lymphocyte ratio and platelet‐lymphocyte ratio in the prognosis of resected early‐stage patients with non‐small cell lung cancer: a meta‐analysis. Cancer Med. 2023;12(6):7065‐7076. 36480232 10.1002/cam4.5505 PMC10067053

[198] Guo G , Hu X , Gao T , et al. Potential impact of platelet‐to‐lymphocyte ratio on prognosis in patients with colorectal cancer: a systematic review and meta‐analysis. Front Surg. 2023;10:1139503. 37051571 10.3389/fsurg.2023.1139503 PMC10083474

[199] Yodying H , Matsuda A , Miyashita M , et al. Prognostic significance of neutrophil‐to‐lymphocyte ratio and platelet‐to‐lymphocyte ratio in oncologic outcomes of esophageal cancer: a systematic review and meta‐analysis. Ann Surg Oncol. 2016;23(2):646‐654. 26416715 10.1245/s10434-015-4869-5

[200] Zhang Y , Zheng L , Quan L , Du L . Prognostic role of platelet‐to‐lymphocyte ratio in oral cancer: a meta‐analysis. J Oral Pathol Med. 2021;50(3):274‐279. 30681182 10.1111/jop.12832

[201] Alexander N . Reference values of neutrophil‐lymphocyte ratio, platelet‐lymphocyte ratio and mean platelet volume in healthy adults in north central Nigeria. J Blood Lymph. 2016;6(1):1000143.

[202] Jhamb R , Kumar R , Gogoi P , Ranga GS , Kashyap B . Reference values of neutrophil lymphocyte ratio and platelet lymphocyte ratio in healthy adults in a tertiary care center in North India. Bionature. 2020;40(3):44‐52.

[203] Wang Y , Attar BM , Fuentes HE , Jaiswal P , Tafur AJ . Evaluation of the prognostic value of platelet to lymphocyte ratio in patients with hepatocellular carcinoma. J Gastrointest Oncol. 2017;8(6):1065‐1071. 29299368 10.21037/jgo.2017.09.06 PMC5750171

[204] Zheng CF , Liu WY , Zeng FF , et al. Prognostic value of platelet‐to‐lymphocyte ratios among critically ill patients with acute kidney injury. Crit Care. 2017;21(1):238. 28882170 10.1186/s13054-017-1821-z PMC5590135

[205] Zheng J , Cai J , Li H , et al. Neutrophil to lymphocyte ratio and platelet to lymphocyte ratio as prognostic predictors for hepatocellular carcinoma patients with various treatments: a meta‐analysis and systematic review. Cell Physiol Biochem. 2017;44(3):967‐981. 29179180 10.1159/000485396

[206] Perumal R , Rajendran M , Krishnamurthy M , Ganji KK , Pendor SD . Modulation of P‐selection and platelet aggregation in chronic periodontitis: a clinical study. J Indian Soc Periodontol. 2014;18(3):293‐300. 25024540 10.4103/0972-124X.134563 PMC4095619

[207] Khode V , Sindhur J , Kanbur D , Ruikar K , Nallulwar S . Mean platelet volume and other platelet volume indices in patients with stable coronary artery disease and acute myocardial infarction: a case control study. J Cardiovasc Dis Res. 2012;3(4):272‐275. 23233769 10.4103/0975-3583.102694 PMC3516005

[208] Vagdatli E , Gounari E , Lazaridou E , Katsibourlia E , Tsikopoulou F , Labrianou I . Platelet distribution width: a simple, practical and specific marker of activation of coagulation. Hippokratia. 2010;14(1):28‐32. 20411056 PMC2843567

[209] Khandekar MM , Khurana AS , Deshmukh SD , Kakrani AL , Katdare AD , Inamdar AK . Platelet volume indices in patients with coronary artery disease and acute myocardial infarction: an Indian scenario. J Clin Pathol. 2006;59(2):146‐149. 16443728 10.1136/jcp.2004.025387 PMC1860313

[210] Cheng S , Han F , Wang Y , et al. The red distribution width and the platelet distribution width as prognostic predictors in gastric cancer. BMC Gastroenterol. 2017;17(1):163. 29262773 10.1186/s12876-017-0685-7 PMC5738162

[211] Xia W , Chen W , Tu J , Ni C , Meng K . Prognostic value and clinicopathologic features of platelet distribution width in cancer: a meta‐analysis. Med Sci Monit. 2018;24:7130‐7136. 30291788 10.12659/MSM.913040 PMC6187965

[212] Takeuchi H , Noda D , Abe M , et al. Evaluating the platelet distribution width‐to‐Plateletcrit ratio as a prognostic marker for patients with breast cancer. Anticancer Res. 2020;40(7):3947‐3952. 32620636 10.21873/anticanres.14386

[213] Zhang H , Liu L , Fu S , et al. Higher platelet distribution width predicts poor prognosis in laryngeal cancer. Oncotarget. 2017;8(29):48138‐48144. 28624815 10.18632/oncotarget.18306 PMC5564632

[214] Song X , Zhu H , Pei Q , et al. Significance of inflammation‐based indices in the prognosis of patients with non‐metastatic colorectal cancer. Oncotarget. 2017;8(28):45178‐45189. 28423351 10.18632/oncotarget.16774 PMC5542176

[215] Chandrashekar V . Plateletcrit as a screening tool for detection of platelet quantitative disorders. J Hematol. 2013;2(1):22‐26.

[216] Wada H , Dohi T , Miyauchi K , et al. Mean platelet volume and long‐term cardiovascular outcomes in patients with stable coronary artery disease. Atherosclerosis. 2018;277:108‐112. 30195145 10.1016/j.atherosclerosis.2018.08.048

[217] Czerniuk MR , Bartoszewicz Z , Dudzik‐Niewiadomska I , Pilecki T , Górska R , Filipiak KJ . Simple platelet markers: mean platelet volume and congestive heart failure coexistent with periodontal disease. Pilot studies. Cardiol J. 2019;26(3):253‐259. 28714524 10.5603/CJ.a2017.0085 PMC8086677

[218] Ekici B , Erkan AF , Alhan A , Sayın I , Aylı M , Töre HF . Is mean platelet volume associated with the angiographic severity of coronary artery disease? Kardiol Pol. 2013;71(8):832‐838. 24049023 10.5603/KP.2013.0195

[219] Cetin MS , Ozcan Cetin EH , Akdi A , et al. Platelet distribution width and plateletcrit: novel biomarkers of ST elevation myocardial infarction in young patients. Kardiol Pol. 2017;75(10):1005‐1012. 28715073 10.5603/KP.a2017.0135

[220] Zhang F , Chen Z , Wang P , Hu X , Gao Y , He J . Combination of platelet count and mean platelet volume (COP‐MPV) predicts postoperative prognosis in both resectable early and advanced stage esophageal squamous cell cancer patients. Tumour Biol. 2016;37(7):9323‐9331. 26779631 10.1007/s13277-015-4774-3 PMC4990601

[221] Boos CJ , Lip GY . Platelet activation and cardiovascular outcomes in acute coronary syndromes. J Thromb Haemost. 2006;4(12):2542‐2543. 17026647 10.1111/j.1538-7836.2006.02250.x

[222] Aslan S , Demir AR , Demir Y , et al. Usefulness of plateletcrit in the prediction of major adverse cardiac and cerebrovascular events in patients with carotid artery stenosis. Vascular. 2019;27(5):479‐486. 31027469 10.1177/1708538119847898

[223] Sahin F , Yazar E , Yıldız P . Prominent features of platelet count, plateletcrit, mean platelet volume and platelet distribution width in pulmonary tuberculosis. Multidiscip Respir Med. 2012;7(1):38. 23114411 10.1186/2049-6958-7-38 PMC3529701

[224] Akpinar I , Sayin MR , Gursoy YC , et al. Plateletcrit and red cell distribution width are independent predictors of the slow coronary flow phenomenon. J Cardiol. 2014;63(2):112‐118. 24012331 10.1016/j.jjcc.2013.07.010

[225] Şahin F , Aslan AF . Relationship between inflammatory and biological markers and lung cancer. J Clin Med. 2018;7(7):160. 29941786 10.3390/jcm7070160 PMC6069225

[226] Hur JY , Lee HY , Chang HJ , Choi CW , Kim DH , Eo WK . Preoperative plateletcrit is a prognostic biomarker for survival in patients with non‐small cell lung cancer. J Cancer. 2020;11(10):2800‐2807. 32226498 10.7150/jca.41122 PMC7086273

[227] Zhu X , Cao Y , Lu P , et al. Evaluation of platelet indices as diagnostic biomarkers for colorectal cancer. Sci Rep. 2018;8(1):11814. 30087357 10.1038/s41598-018-29293-x PMC6081379

[228] Zhang X , Wu YY , Qin YY , Lin FQ . The combined detection of hematological indicators is used for the differential diagnosis of colorectal cancer and benign‐colorectal lesions. Cancer Biomark. 2023;39:223‐230. 10.3233/CBM-230157 PMC11091605 38217586

[229] Zhao X , Yang Y , Pan Z , et al. Plateletcrit is predictive of clinical outcome and prognosis for early‐stage breast cancer: a retrospective cohort study based on propensity score matching. Cancer Med. 2024;13(2):e6944. 38348939 10.1002/cam4.6944 PMC10832319

[230] Kisa E , Yucel C , Keskin MZ , et al. The role of hematological parameters in predicting Fuhrman grade and tumor stage in renal cell carcinoma patients undergoing nephrectomy. Medicina (Kaunas). 2019;55(6):287. 31216752 10.3390/medicina55060287 PMC6630220

[231] Wang L , Sheng L , Liu P . The independent association of platelet parameters with overall survival in pancreatic adenocarcinoma receiving intensity‐modulated radiation therapy. Int J Clin Exp Med. 2015;8(11):21215‐21221. 26885057 PMC4723902

[232] Hu B , Yang XR , Xu Y , et al. Systemic immune‐inflammation index predicts prognosis of patients after curative resection for hepatocellular carcinoma. Clin Cancer Res. 2014;20(23):6212‐6222. 25271081 10.1158/1078-0432.CCR-14-0442

[233] Qin Z , Li H , Wang L , et al. Systemic immune‐inflammation index is associated with increased urinary albumin excretion: a population‐based study. Front Immunol. 2022;13:863640. 35386695 10.3389/fimmu.2022.863640 PMC8977553

[234] Meng L , Yang Y , Hu X , Zhang R , Li X . Prognostic value of the pretreatment systemic immune‐inflammation index in patients with prostate cancer: a systematic review and meta‐analysis. J Transl Med. 2023;21(1):79. 36739407 10.1186/s12967-023-03924-y PMC9898902

[235] Luo H , He L , Zhang G , et al. Normal reference intervals of neutrophil‐to‐lymphocyte ratio, platelet‐to‐lymphocyte ratio, lymphocyte‐to‐monocyte ratio, and systemic immune inflammation index in healthy adults: a large multi‐center study from Western China. Clin Lab. 2019;65(3). 10.7754/Clin.Lab.2018.180715 30868857

[236] Fei Y , Wang X , Zhang H , Huang M , Chen X , Zhang C . Reference intervals of systemic immune‐inflammation index, neutrophil to lymphocyte ratio, platelet to lymphocyte ratio, mean platelet volume to platelet ratio, mean platelet volume and red blood cell distribution width‐standard deviation in healthy Han adults in Wuhan region in central China. Scand J Clin Lab Invest. 2020;80(6):500‐507. 32673141 10.1080/00365513.2020.1793220

[237] Fest J , Ruiter R , Ikram MA , Voortman T , van Eijck CHJ , Stricker BH . Reference values for white blood‐cell‐based inflammatory markers in the Rotterdam study: a population‐based prospective cohort study. Sci Rep. 2018;8(1):10566. 30002404 10.1038/s41598-018-28646-w PMC6043609

[238] Dincer Rota D , Tanacan E . The utility of systemic‐immune inflammation index for predicting the disease activation in patients with psoriasis. Int J Clin Pract. 2021;75(6):e14101. 33619821 10.1111/ijcp.14101

[239] Xia Y , Xia C , Wu L , Li Z , Li H , Zhang J . Systemic immune inflammation index (SII), system inflammation response index (SIRI) and risk of all‐cause mortality and cardiovascular mortality: a 20‐year follow‐up cohort study of 42,875 US adults. J Clin Med. 2023;12(3):1128. 36769776 10.3390/jcm12031128 PMC9918056

[240] Wang YT , Kuo LT , Weng HH , et al. Systemic Immun e‐inflammation index as a predictor for head and neck cancer prognosis: a meta‐analysis. Front Oncol. 2022;12:899518. 35814369 10.3389/fonc.2022.899518 PMC9263088

[241] Ji Y , Wang H . Prognostic prediction of systemic immune‐inflammation index for patients with gynecological and breast cancers: a meta‐analysis. World J Surg Oncol. 2020;18(1):197. 32767977 10.1186/s12957-020-01974-w PMC7414550

[242] Zhou Y , Dai M , Zhang Z . Prognostic significance of the systemic immune‐inflammation index (SII) in patients with small cell lung cancer: a meta‐analysis. Front Oncol. 2022;12:814727. 35186750 10.3389/fonc.2022.814727 PMC8854201

[243] Li M , Li Z , Wang Z , Yue C , Hu W , Lu H . Prognostic value of systemic immune‐inflammation index in patients with pancreatic cancer: a meta‐analysis. Clin Exp Med. 2022;22(4):637‐646. 35022918 10.1007/s10238-021-00785-x

[244] Qiu Y , Zhang Z , Chen Y . Prognostic value of pretreatment systemic immune‐inflammation index in gastric cancer: a meta‐analysis. Front Oncol. 2021;11:537140. 33777726 10.3389/fonc.2021.537140 PMC7990885

[245] Nøst TH , Alcala K , Urbarova I , et al. Systemic inflammation markers and cancer incidence in the UK Biobank. Eur J Epidemiol. 2021;36(8):841‐848. 34036468 10.1007/s10654-021-00752-6 PMC8416852

[246] Liu B , Wang J , Li YY , Li KP , Zhang Q . The association between systemic immune‐inflammation index and rheumatoid arthritis: evidence from NHANES 1999‐2018. Arthritis Res Ther. 2023;25(1):34. 36871051 10.1186/s13075-023-03018-6 PMC9985219

[247] Hajishengallis G . New developments in neutrophil biology and periodontitis. Periodontol 2000. 2020;82(1):78‐92. 31850633 10.1111/prd.12313

[248] Nascimento GG , Leite FRM , Vestergaard P , Scheutz F , López R . Does diabetes increase the risk of periodontitis? A systematic review and meta‐regression analysis of longitudinal prospective studies. Acta Diabetol. 2018;55(7):653‐667. 29502214 10.1007/s00592-018-1120-4

[249] Yang YL , Wu CH , Hsu PF , et al. Systemic immune‐inflammation index (SII) predicted clinical outcome in patients with coronary artery disease. Eur J Clin Invest. 2020;50(5):e13230. 32291748 10.1111/eci.13230

[250] Xu JP , Zeng RX , Zhang YZ , et al. Systemic inflammation markers and the prevalence of hypertension: a NHANES cross‐sectional study. Hypertens Res. 2023;46(4):1009‐1019. 36707716 10.1038/s41440-023-01195-0

[251] de Oliveira FR , de Brito SR , Magno MB , et al. Does periodontitis represent a risk factor for rheumatoid arthritis? A systematic review and meta‐analysis. Ther Adv Musculoskelet Dis. 2019;11:1759720X19858514. 10.1177/1759720X19858514 PMC6620730 31316593

[252] Zhang X , Gu H , Xie S , Su Y . Periodontitis in patients with psoriasis: a systematic review and meta‐analysis. Oral Dis. 2022;28(1):33‐43. 32852860 10.1111/odi.13617 PMC9290533

[253] Mishra S , Johnson L , Agrawal S , Rajput S . Assessment of periodontal status in patients with psoriatic arthritis: a retrospective, case‐control study. J Clin Exp Dent. 2021;13(8):e776‐e783. 34512916 10.4317/jced.58125 PMC8412814

[254] Johnson CL , Dohrmann SM , Burt VL , Mohadjer LK . National health and nutrition examination survey: sample design, 2011‐2014. Vital Health Stat 2. 2014;162:1‐33. 25569458

[255] Bamashmous S , Kotsakis GA , Kerns KA , et al. Human variation in gingival inflammation. Proc Natl Acad Sci USA. 2021;118(27):e2012578118. 34193520 10.1073/pnas.2012578118 PMC8271746

[256] Liu YY , Ruan GT , Ge YZ , et al. Systemic inflammation with sarcopenia predicts survival in patients with gastric cancer. J Cancer Res Clin Oncol. 2023;149(3):1249‐1259. 35435489 10.1007/s00432-022-03925-2 PMC11796546

[257] Balta MG , Papathanasiou E , Blix IJ , Van Dyke TE . Host modulation and treatment of periodontal disease. J Dent Res. 2021;100(8):798‐809. 33655803 10.1177/0022034521995157 PMC8261853

[258] Golub LM , Lee HM . Periodontal therapeutics: current host‐modulation agents and future directions. Periodontol 2000. 2020;82(1):186‐204. 31850625 10.1111/prd.12315 PMC6973248

[259] Farmer HR , Slavish DC , Ruiz J , et al. Racial/ethnic variations in inflammatory markers: exploring the role of sleep duration and sleep efficiency. J Behav Med. 2022;45(6):855‐867. 36029411 10.1007/s10865-022-00357-8 PMC10062430

[260] Evans TC , Jehle D . The red blood cell distribution width. J Emerg Med. 1991;9(Suppl 1):71‐74. 1955687 10.1016/0736-4679(91)90592-4

[261] McClure S , Custer E , Bessman JD . Improved detection of early iron deficiency in nonanemic subjects. JAMA. 1985;253(7):1021‐1023. 3968826

[262] Harrington AM , Ward PC , Kroft SH . Iron deficiency anemia, beta‐thalassemia minor, and anemia of chronic disease: a morphologic reappraisal. Am J Clin Pathol. 2008;129(3):466‐471. 18285271 10.1309/LY7YLUPE7551JYBG

[263] Wians FH Jr , Urban JE , Keffer JH , Kroft SH . Discriminating between iron deficiency anemia and anemia of chronic disease using traditional indices of iron status vs transferrin receptor concentration. Am J Clin Pathol. 2001;115(1):112‐118. 11190796 10.1309/6L34-V3AR-DW39-DH30

[264] Thompson WG , Meola T , Lipkin M Jr , Freedman ML . Red cell distribution width, mean corpuscular volume, and transferrin saturation in the diagnosis of iron deficiency. Arch Intern Med. 1988;148(10):2128‐2130. 3178371

[265] Nah EH , Kim S , Cho S , Cho HI . Complete blood count reference intervals and patterns of changes across pediatric, adult, and geriatric ages in Korea. Ann Lab Med. 2018;38(6):503‐511. 30027692 10.3343/alm.2018.38.6.503 PMC6056383

[266] Cavusoglu E , Chopra V , Gupta A , et al. Relation between red blood cell distribution width (RDW) and all‐cause mortality at two years in an unselected population referred for coronary angiography. Int J Cardiol. 2010;141(2):141‐146. 19144426 10.1016/j.ijcard.2008.11.187

[267] Perlstein TS , Weuve J , Pfeffer MA , Beckman JA . Red blood cell distribution width and mortality risk in a community‐based prospective cohort. Arch Intern Med. 2009;169(6):588‐594. 19307522 10.1001/archinternmed.2009.55 PMC3387573

[268] Tonelli M , Sacks F , Arnold M , Moye L , Davis B , Pfeffer M . Relation between red blood cell distribution width and cardiovascular event rate in people with coronary disease. Circulation. 2008;117(2):163‐168. 18172029 10.1161/CIRCULATIONAHA.107.727545

[269] Bujak K , Wasilewski J , Osadnik T , et al. The prognostic role of red blood cell distribution width in coronary artery disease: a review of the pathophysiology. Dis Markers. 2015;2015:824624. 26379362 10.1155/2015/824624 PMC4563066

[270] Kaya A , Tukkan C , Alper AT , et al. Increased levels of red cell distribution width is correlated with presence of left atrial stasis in patients with non‐valvular atrial fibrillation. North Clin Istanb. 2017;4(1):66‐72. 28752145 10.14744/nci.2017.72324 PMC5530160

[271] Yin JM , Zhu KP , Guo ZW , Yi W , He Y , Du GC . Is red cell distribution width a prognostic factor in patients with breast cancer? A meta‐analysis. Front Surg. 2023;10:1000522. 37035565 10.3389/fsurg.2023.1000522 PMC10079877

[272] Wang DP , Kang K , Lin Q , Hai J . Prognostic significance of preoperative systemic cellular inflammatory markers in gliomas: a systematic review and meta‐analysis. Clin Transl Sci. 2020;13(1):179‐188. 31550075 10.1111/cts.12700 PMC6951460

[273] Wang Y , Zhou Y , Zhou K , Li J , Che G . Prognostic value of pre‐treatment red blood cell distribution width in lung cancer: a meta‐analysis. Biomarkers. 2020;25(3):241‐247. 32064949 10.1080/1354750X.2020.1731763

[274] Xu WY , Yang XB , Wang WQ , et al. Prognostic impact of the red cell distribution width in esophageal cancer patients: a systematic review and meta‐analysis. World J Gastroenterol. 2018;24(19):2120‐2129. 29785080 10.3748/wjg.v24.i19.2120 PMC5960817

[275] Yan S , Kong J , Zhao ZF , Yao H . The prognostic importance of red blood cell distribution width for gastric cancer: a systematic review and meta‐analysis. Transl Cancer Res. 2023;12(7):1816‐1825. 37588748 10.21037/tcr-23-53 PMC10425649

[276] Wen ZL , Zhou X , Xiao DC . Is red blood cell distribution width a prognostic factor for colorectal cancer? A meta‐analysis. Front Surg. 2022;9:945126. 36263092 10.3389/fsurg.2022.945126 PMC9574073

[277] Cao W , Shao Y , Wang N , Jiang Z , Yu S , Wang J . Pretreatment red blood cell distribution width may be a potential biomarker of prognosis in urologic cancer: a systematic review and meta‐analysis. Biomark Med. 2022;16(18):1289‐1300. 36912229 10.2217/bmm-2022-0409

[278] Wang PF , Song SY , Guo H , Wang TJ , Liu N , Yan CX . Prognostic role of pretreatment red blood cell distribution width in patients with cancer: a meta‐analysis of 49 studies. J Cancer. 2019;10(18):4305‐4317. 31413750 10.7150/jca.31598 PMC6691718

[279] Beck JD , Offenbacher S . The association between periodontal diseases and cardiovascular diseases: a state‐of‐the‐science review. Ann Periodontol. 2001;6(1):9‐15. 11887476 10.1902/annals.2001.6.1.9

[280] Blaizot A , Vergnes JN , Nuwwareh S , Amar J , Sixou M . Periodontal diseases and cardiovascular events: meta‐analysis of observational studies. Int Dent J. 2009;59(4):197‐209. 19774803

[281] Pradeep AR , Anuj S . Anemia of chronic disease and chronic periodontitis: does periodontal therapy have an effect on anemic status? J Periodontol. 2011;82(3):388‐394. 20843237 10.1902/jop.2010.100336

[282] Sun XJ , Meng HX , Shi D , et al. Elevation of C‐reactive protein and interleukin‐6 in plasma of patients with aggressive periodontitis. J Periodontal Res. 2009;44(3):311‐316. 18842114 10.1111/j.1600-0765.2008.01131.x

[283] Cairo F , Nieri M , Gori AM , et al. Markers of systemic inflammation in periodontal patients: chronic versus aggressive periodontitis. An explorative cross‐sectional study. Eur J Oral Implantol. 2010;3(2):147‐153. 20623039

[284] Larvin H , Kang J , Aggarwal VR , Pavitt S , Wu J . Systemic multimorbidity clusters in people with periodontitis. J Dent Res. 2022;101(11):1335‐1342. 35678074 10.1177/00220345221098910 PMC9516606

[285] Beukers NGFM , Su N , van der Heijden GJMG , Loos BG . Periodontitis is associated with multimorbidity in a large dental school population. J Clin Periodontol. 2023;50(12):1621‐1632. 37658672 10.1111/jcpe.13870

[286] Wu X , Zhao M , Pan B , et al. Complete blood count reference intervals for healthy Han Chinese adults. PLoS One. 2015;10(3):e0119669. 25769040 10.1371/journal.pone.0119669 PMC4358890

[287] Leite FRM , Nascimento GG , Scheutz F , López R . Effect of smoking on periodontitis: a systematic review and meta‐regression. Am J Prev Med. 2018;54(6):831‐841. 29656920 10.1016/j.amepre.2018.02.014

[288] Varol E , Ozaydin M . Mean platelet volume in patients with acute pancreatitis: insight from methodological aspect. Blood Coagul Fibrinolysis. 2014;25(2):196‐197. 10.1097/MBC.0b013e328364e42a 24477229

[289] Gasparyan AY , Ayvazyan L , Mikhailidis DP , Kitas GD . Mean platelet volume: a link between thrombosis and inflammation? Curr Pharm Des. 2011;17(1):47‐58. 21247392 10.2174/138161211795049804

[290] Varol E , Ozaydin M . Confounding factors should be considered in the evaluation of mean platelet volume in nonvalvular atrial fibrillation. Blood Coagul Fibrinolysis. 2015;26(2):230. 25629418 10.1097/MBC.0000000000000201

[291] Bartold PM , Mariotti A . The future of periodontal‐systemic associations: raising the standards. Curr Oral Health Rep. 2017;4(3):258‐262. 28944159 10.1007/s40496-017-0150-2 PMC5587612

[292] Cornet E , Boubaya M , Troussard X . Contribution of the new XN‐1000 parameters NEUT‐RI and NEUT‐WY for managing patients with immature granulocytes. Int J Lab Hematol. 2015;37(5):e123‐e126. 25923650 10.1111/ijlh.12372

[293] Henriot I , Launay E , Boubaya M , et al. New parameters on the hematology analyzer XN‐10 (SysmexTM) allow to distinguish childhood bacterial and viral infections. Int J Lab Hematol. 2017;39(1):14‐20. 10.1111/ijlh.12562 27572612

[294] Oehadian A , Michels M , de Mast Q , et al. New parameters available on Sysmex XE‐5000 hematology analyzers contribute to differentiating dengue from leptospirosis and enteric fever. Int J Lab Hematol. 2015;37(6):861‐868. 26333341 10.1111/ijlh.12422

